# Evolution and Application of Inteins in *Candida* species: A Review

**DOI:** 10.3389/fmicb.2016.01585

**Published:** 2016-10-10

**Authors:** José A. L. Fernandes, Tâmara H. R. Prandini, Maria da Conceiçao A. Castro, Thales D. Arantes, Juliana Giacobino, Eduardo Bagagli, Raquel C. Theodoro

**Affiliations:** ^1^Institute of Tropical Medicine of Rio Grande do Norte, Universidade Federal do Rio Grande do NorteNatal, Brazil; ^2^Department of Microbiology and Immunology, Institute of Biosciences, Universidade Estadual Paulista Julio de Mesquita FilhoBotucatu, Brazil; ^3^Post-graduation Program in Biochemistry, Universidade Federal do Rio Grande do NorteNatal, Brazil

**Keywords:** *Candida* spp., intein, vacuolar ATPase, threonyl-tRNA synthetase, glutamate syntethase, molecular identification, new therapeutic targets

## Abstract

Inteins are invasive intervening sequences that perform an autocatalytic splicing from their host proteins. Among eukaryotes, these elements are present in many fungal species, including those considered opportunistic or primary pathogens, such as *Candida* spp. Here we reviewed and updated the list of *Candida* species containing inteins in the genes *VMA, THRRS* and *GLT1* and pointed out the importance of these elements as molecular markers for molecular epidemiological researches and species-specific diagnosis, since the presence, as well as the size of these inteins, is polymorphic among the different species. Although absent in *Candida albicans*, these elements are present in different sizes, in some environmental *Candida* spp. and also in most of the non-albicans *Candida* spp. considered emergent opportunistic pathogens. Besides, the possible role of these inteins in yeast physiology was also discussed in the light of the recent findings on the importance of these elements as post-translational modulators of gene expression, reinforcing their relevance as alternative therapeutic targets for the treatment of non-albicans *Candida* infections, because, once the splicing of an intein is inhibited, its host protein, which is usually a housekeeping protein, becomes non-functional.

## Introduction

Inteins are invasive genetic elements that occur as intervening sequences in conserved coding host genes. They are transcribed and translated with the flanking host protein sequences and then self-excised by protein splicing. The flanking protein sequences (exteins) are joined by a peptide bond, constituting the functional protein (Chong et al., 1996; Perler, 2005).

Over the past three decades, inteins have been detected mainly in unicellular microorganisms in the three domains of life and in viruses (Perler, 2002). Among the Eukarya domain, inteins are found mostly in fungi, some green algae and other basal eukaryotes (Liu, 2000; Butler et al., 2001, 2006). In a recent review, 2729 genomes of bacteria, 345 of archaea and 6648 of eukarya were analyzed (Topilina et al., 2015b) and 24, 47, and 1.1% of these genomes, respectively, presented at least one intein.

Inteins are usually found at conserved sites of housekeeping proteins that have vital functions in the cell, such as DNA and RNA polymerases, aminoacyl tRNA synthetases, recombinases, topoisomerases, helicases and essential components of the spliceosome (Novikova et al., 2014). Some hypothesis for this distribution already exist and are in part supported by some evolutionary scenarios, including probable horizontal transfer, as well as genetic mobility of inteins by homing endonuclease, which means that the spread and increase of this element in populations is due to a gene conversion process by homologous recombination, rather than any selective advantage (Nagasaki et al., 2005; Gogarten and Hilario, 2006; Swithers et al., 2009). This idea rendered to inteins the title of “parasitic” genetic elements during the past 25 years, although some domesticated inteins, such as HO gene of *Saccharomyces cerevisiae* showed to have “gained” a function in the cell biology. In this specific case, the HO gene encodes for an endonuclease responsible for mating type conversion in yeast and its splicing domain is not active anymore (Koufopanou and Burt, 2005).

Nevertheless, recent works have evidenced a possible function for inteins in the post-translational regulation of gene expression. For instance, the splicing of SufB intein of *Mycobacterium tuberculosis* showed to be inhibited by reactive oxygen and nitrogen species (ROS and RNS) when expressed in *Escherichia coli*. These stressful conditions are also experimented by *M. tuberculosis* inside the macrophage (Topilina et al., 2015a). Other evidence came from the splicing modulation of RadA intein from the hyperthermophilic archaeon *Pyrococcus horikoshii* according to the temperature, solution conditions and remote extein point mutations. So, RadA intein might function as an environmental sensor, releasing the intein for full activity only at optimal growth conditions for the native organism (high temperatures), while sparing ATP consumption under cold-shock. The authors observed intein splicing at low temperature only after adding a combination of the detergent SDS and the ionic liquid 1-butyl-3-methylimidazolium chloride, showing that, besides temperature, other factors may also interfere in RadA intein splicing (Topilina et al., 2015b).

Although the experimental evidence for the intein’s role in modulation of gene expression is based on a non-native context (different extein and different host cell), it is an important clue for the actual functionality of these elements in nature. Their presence in particular conserved motifs might be explained by an adaptive process (Novikova et al., 2014). Novikova et al. (2015) observed that inteins have a certain “preference” for specific functional domains of related housekeeping proteins, like ATPase domains for example, and this does not entirely fit to the models describing inteins as merely mobile parasitic elements. The authors argue that this intein distribution might be a result of a selective retention of these elements, which might be beneficial under certain environmental stresses. So, the sporadic nature of intein in closely related species could be explained by different environmental stresses. If there is no strong selective pressure for intein maintenance in a certain subpopulation and its presence reduces the adaptive value of the host microorganism, the intein-free alleles will increase in this population by means of natural selection (Gogarten and Hilario, 2006).

Nevertheless, we are far from a global understanding about the reason for intein persistence in different housekeeping host proteins, only in unicellular organisms, over millions of years. It seems consensual that their maintenance may be due to either their parasitic nature, since they are invasive elements, as well as to a possible role in specific physiological conditions of the host cell. Yet, among the mobile elements, inteins are the least studied, so that few inteins have already had their activity tested under different conditions; and therefore their real function is still a puzzle. Regarding that most of the produced knowledge on genome organization during the last years has been changing the status of many mobile genes, mainly retrotransposable elements, which are intensively studied, from parasitic entities to dynamic elements involved in genome evolution and gene expression (Mita and Boeke, 2016), it seems plausible that intein’s function in cell deserves more scientific investigation. The more we understand about inteins, the better we can explore their biotechnological applicability.

Few fungal inteins have been described, most of them are located in *PRP8* (precursor mRNA protein) and *VMA* (vacuolar ATPase) coding genes, others can be observed in some RNA Polymerase subunits, *GLT1* (glutamate synthase), *CHS2* (chitin synthase) and *THRRS* (threonyl-tRNA synthetase) coding genes (Poulter et al., 2007). Most fungal inteins are present in the *PRP8* and *VMA* genes. The PRP8 intein is widely dispersed in Fungi Kingdom (fungi containing this intein are found in different phyla), being present in many important human pathogens, such as *Cryptococcus neoformans, Cryptococcus gattii, Histoplasma capsulatum, Emmonsia* spp., *Paracoccidioides* spp., *Blastomyces dermatitidis, Aspergillus nidulans, Aspergillus fumigatus* (Liu and Yang, 2004; Butler and Poulter, 2005; Butler et al., 2006; Theodoro et al., 2010). The VMA intein, on the other hand, is specifically distributed among Saccharomycetes, such as *Saccharomyces, Zygosacchromyces* and some *Candida*.

Here we updated the information on the intein distribution among *Candida* species and explored their potential as a source of phylogenetic information and, therefore, as species-specific diagnostic tool. Besides, the possible impact of the presence of these elements in housekeeping genes in *Candida* species life style was discussed raising their promising application as drug targets for most of the medically relevant non-albicans emergent *Candida* species.

## Types of Inteins and How They Operate

**FIGURE 1:**
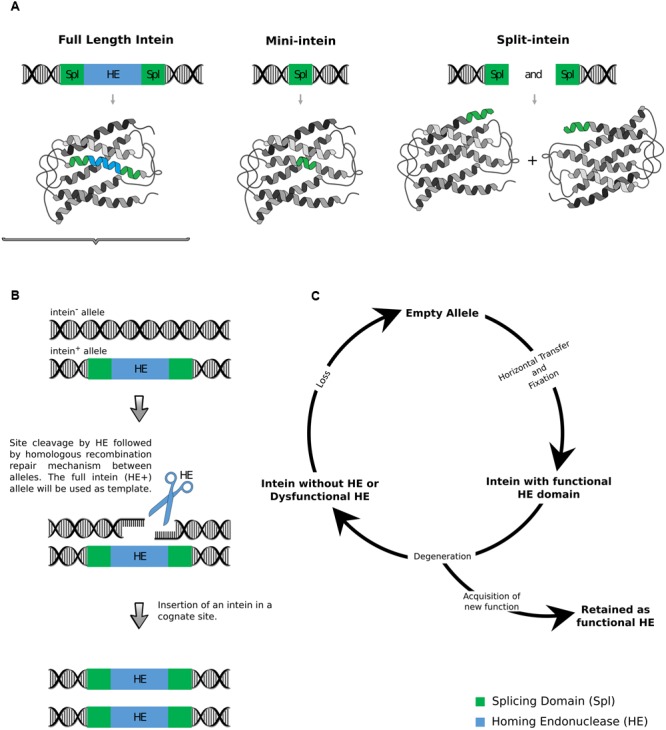
**(A)** Full length, mini and split inteins. **(B)** Intein mobility: DSB by HE and homologous recombination repair. **(C)** Cycle model of HE gain, degeneration, and loss within host populations modified from Burt and Koufopanou (2004).

Inteins come in three configurations: full-length, mini and split (**Figure 1A**). The full-length inteins have a homing endonuclease domain (HE) splitting the splicing domain in N and C-Spl terminals. When active, the HE recognizes a cognate allele, without the intein, performs a double strand break (DSB) and, by homologous recombination, copies the intein using the intein-containing allele as template for DNA repair. Mini-inteins lack the HE domain and have a continuous splicing domain, while split-inteins are mini-inteins, whose N and C-Spl terminals are transcribed and translated with different exteins. When the N and C-Spl terminals are assembled, the intein suffers a *trans*-splicing reaction, ligating the different exteins (Wood et al., 1999; Volkmann and Iwaı, 2010).

Intein mobility is triggered by its HE domain which recognizes long, and therefore specific, DNA sequences (14–40 bp) lacking the intein, producing a DSB. The host repair system is then activated and the template containing the intein is “copied” to the recipient chromosome by a gene conversion event (**Figure 1B**), distorting the Mendelian segregation, causing a Super-Mendelian inheritance or a rapid dissemination and fixation of the intein in population. When all alleles of a certain population are occupied by the intein there is no selection pressure for functional HE, because both, functional and non-functional HEs will be equally maintained in population simply by cellular propagation and the HE domain is “free” to degenerate (**Figure 1C**). That is why the HE domain is expected to have more sequence variation, including its complete degeneration and/or deletion, than the splicing domain (Burt and Koufopanou, 2004; Koufopanou and Burt, 2005; Gogarten and Hilario, 2006).

Inteins are particularly interesting as a source of phylogenetic information because they present the well-preserved splicing domain (N and C terminals), with the conserved motifs A, B (for the N-splicing) and F, G (for the C-splicing), flanking the central and more variable HE domain, in the full-length inteins. The presence of the motifs C, D, E, and H, as well as the two aspartic acid residues, one in C and other in E, in HEs from LAGLIDADG family (the most common in fungal inteins), indicate that the HE domain is probably active (Gimble and Thorner, 1992; Liu, 2000; Posey et al., 2004). In mini-inteins, HE domains are supposed to be completely deleted. In addition, their location in highly conserved genes facilitates the design of primers for their amplification (Butler and Poulter, 2005; Theodoro and Bagagli, 2009; Prandini et al., 2013).

Besides its potential as molecular markers, inteins have been largely used for biotechnological purposes, accordingly to their functional mechanisms. The splicing domain, for example, is explored for purification of recombinant proteins, cyclization of proteins, protein labeling, production of selenoproteins (Miraula et al., 2015) and, for pathogenic microorganisms, it has been studied as a drug target (Paulus, 2007; Zhang et al., 2010). The HE domain has also been intensively studied as a biotechnological tool, in this case for genome editing, since these enzymes can be reprogrammed to specifically cut a genome site and deliver a gene of interest by homologous recombination. The applications of this technology are numerous for different social-economic fields, such as gene therapy for monogenic diseases, insect vector control (already developed for malaria mosquitoes) and gene delivery for transgenic plants, such as maize (Belfort and Bonocora, 2014).

## Inteins in *Candida* Species

*Candida* species belong to Ascomycota phylum, Hemiascomycetes class and Saccharomycetales order. These species are distributed in three different clades making *Candida* a non-monophyletic group (Diezmann et al., 2004). With the exception of *C. glabrata*, which is in clade 3, together with *C. castelli, C. norvegica, Saccharomyces cerevisiae* and other biotechnologically important yeast species, the most relevant clinical species of *Candida* are clustered in clade 1, which includes *C. albicans, C. parapsilosis, C. orthopsilosis, C. metapsilosis, C. tropicalis*, and *C. viswanathii*, as well as two non-pathogenic species, *Candida maltosa* and *Lodderomyces elongisporus*. *C. guilliermondii, C. intermedia, C. famata*, and *C. zeylanoides* are clustered in clade 2. The clades 1 and 2 are also referred as CUG group by distinctly translate this codon as serine instead of the standard leucine. Whether this attribute may play a hole in pathogenicity is still unknown (Diezmann et al., 2004).

*Candida* spp. can colonize human tissues in different ways, varying from a normal microbiota in the intestinal mucosa, contiguously to the vaginal mucosa, to a true pathogen. The transition to pathogenicity in *Candida* species, mainly in *C. albicans*, occurs generally due to the host immunological condition, leading to a microbiota unbalance, which associated with important virulence factors, such as biofilm formation and hydrolytic enzymes production, promotes fungal dissemination to other sites and organs. The clinical manifestations vary from a localized mucosa or skin infection to disseminated disease (Lagunes and Rello, 2016).

Although less common, disseminated candidiasis has significantly increased over the last 15 years, affecting mainly patients under debilitating and immunosuppressive conditions. Data from Centers for Disease Control and Prevention (CDC) and the National Healthcare Safety Network, show that *Candida* species are the fifth pathogen related to nosocomial diseases and fourth pathogen among bloodstream infections – BSI (Wisplinghoff et al., 2004; Pfaller and Diekema, 2007). These nosocomial *Candida* infections are related to increment of invasive procedures and also to the intensive use of broad-spectrum antimicrobials, particularly in patients admitted in Intensive Care Units – ICU (Colombo et al., 2013).

About 95% of diagnosed fungal infections are related to *Candida* spp., mainly involving the species *C. albicans, C. glabrata, C. tropicalis, C. parapsilosis, C. krusei, C. guilliermondii, C. lusitaniae, C. dubliniensis, C. pelliculosa, C. kefyr, C. lipolytica, C. famata, C. inconspicua, C. rugosa, and C. norvegensis*, but *C. albicans* is still the most frequent species causing infection (Yapar, 2014). However, *Candida* infections caused by non-albicans *Candida* spp. have been increasing in the last few years (Richardson and Lass-Flörl, 2008). In a recent epidemiological analysis, non-albicans species accounted for more than 50% of all cases of invasive candidiasis in patients enrolled in the Prospective Antifungal Therapy Alliance, a sentinel surveillance network comprising 23 medical centers in the US and two in Canada. The non-albicans species identified were *C. glabrata* (46.4%), *C. parapsilosis* (24.7%), *C. tropicalis* (13.9%), *C. krusei* (5.5%), *C. lusitaniae* (1.6%), *C. dubliniensis* (1.5%) and *C. guilliermondii* (0.4%) (Pfaller et al., 2014). While *C. albicans* infections have been progressively replaced by *C. glabrata* infections in North America and in many European countries, *C. parapsilosis* or *C. tropicalis* are the most frequent non-albicans species in Latin America (Nucci et al., 2010; Cleveland et al., 2012; Lockhart et al., 2012). The central factor for the emergence of these non-albicans *Candida* spp. seems to be the undiscriminating use of antifungal drugs (Yapar, 2014).

The treatment of *Candida* infections, mainly candidemias, is based on antifungal agents that interfere on different metabolic pathways being fungicide, such as polyenes, azoles and equinocandis, or fungistatic, such as nucleoside analogs (Lopez-Martinez, 2010; Maubon et al., 2014). The polyenes act on the ergosterol, increasing the permeability of cell membrane by producing aqueous pores; the azoles inhibit the ergosterol biosynthesis and the echinocandins inhibit the synthesis of 1,3 β-glucan, a polysaccharide responsible for the cell wall integrity; and the nucleoside analogs inhibit DNA synthesis being used in combination with amphotericin B or fluconazole (Spampinato and Leonardi, 2013; Paramythiotou et al., 2014), which are the usual chosen drugs for treatment of *Candida* infections (Paramythiotou et al., 2014). The antifungal susceptibility can be easily accessed by Clinical and Laboratory Standards Institute (CLSI) reference microdilution method and also by commercially available antifungal susceptibility testing systems, such as the Sensititre YeastOne colorimetric panel (TREK Diagnostic Systems, Cleveland, OH, USA) (Pfaller et al., 2012).

Monitoring changes in antifungal drug resistance is as important as determining the incidence of candidemia by different *Candida* species. A recent report of surveillance for candidemia in Atlanta, GA, USA and Baltimore, MD, USA over a 5-year period, showed a shift in the species distribution among causative organisms, with a significant increase in *C. glabrata*, as well as in its resistance to echinocandin and fluconazole (Wang et al., 2012; Cleveland et al., 2015). This emergence of antifungal resistance, due to inappropriate prescriptions (Sardi et al., 2013), and the usual toxic side effects of some of these drugs strengthen the demand for new therapeutic targets.

The fungal systemic disease caused by *Candida* is an important cause of mortality, mainly in nosocomial infections and, since the antifungal susceptibility may vary among the different species (Bassetti et al., 2015), the correct diagnosis at species level is necessary. This can be accomplished by classical phenotypic methods, biochemical and physiological tests, which have automated versions, such as Vitek 2 ID – YST (bioMerieux), (Higashi et al., 2015). However, molecular epidemiological studies using different molecular markers (25S group I intron, rDNA D1/D2 region and ITS1-5.8S-ITS2) have revealed intraspecific variation in *C. albicans* and non-albicans *Candida* species and its correlation to antifungal susceptibility (Miletti and Leibowitz, 2000; Karahan et al., 2004; Steuer et al., 2004; Fahami et al., 2010; Gurbuz and Kaleli, 2010; Merseguel et al., 2015).

Besides intraspecific variation, molecular markers have also pointed out the existence of cryptic species in *C. parapsilosis*, actually composed by three species: *C. parapsilosis, C. metapsilosis* and *C. orthopsilosis* (Tavanti et al., 2005), which present differences in virulence and antifungal response. *C. parapsilosis* and *C. orthopsilosis* are more virulent in reconstituted human tissues models and the minimal inhibitory concentration values (MIC) of amphotericin B, caspofungin, anidulafungin and micafungin for *C. orthopsilosis* and *C. metapsilosis* isolates were significantly lower than those for *C. parapsilosis* (Gacser et al., 2007; Lockhart et al., 2008).

The correct recognition of close fungal genotypes or cryptic species would improve the treatment; however, this is not achieved by the usual biochemical methods available in most routine laboratories, mainly in developing countries. The distinction of these very close *Candida* species requires molecular techniques such as PCR- RFLP of *SADH* gene (Tavanti et al., 2005), quantitative PCR (qPCR) (Hays et al., 2011; Souza et al., 2012), pyrosequencing (Borman et al., 2009), microsatellite analysis (Lasker et al., 2006) or matrix-assisted laser desorption ionization–time (MALDI-TOF MS analysis) (Quiles-Melero et al., 2012). This last technique is based on the mass spectrum analysis of crude protein cell extract and requires validated databases for the achievement of rapid and reliable pathogen identification. Recent researches have created databases for the identification of bloodstream yeasts and showed practically 100% accuracy in distinguishing medically important species, such as *C. tropicalis, C. parapsilosis, C. pelliculosa, C. orthopsilosis, C. albicans, C. rugosa, C. guilliermondii, C. lipolytica, C. metapsilosis, C. nivariensis* (De Carolis et al., 2014; Ghosh et al., 2015). The main apparent disadvantage of this technology is the up-front cost of purchasing a MALDI-TOF MS instrument; however, it is offset in about 3 years, providing a noteworthy long-term cost saving for the laboratory (Tran et al., 2015). Some authors have also pointed out the relatively low analytical sensitivity of the method, as well as the few advances for distinguishing filamentous fungi, when compared to the yeast databases (Bailey et al., 2013).

In this scenario, intein research is particularly interesting because they might be a valuable additional or alternative molecular markers for species identification, such as observed for *Cryptococcus* spp., *Paracoccidioides* spp., *Histoplasma capsulatum*, and *Candida* spp. (Butler and Poulter, 2005; Theodoro et al., 2008; Prandini et al., 2013; Theodoro et al., 2013; Satish Kumar and Ramesh, 2014) and also, since they are usually present in housekeeping genes and absent in multicellular eukaryotes, they can be explored as drug targets, because protein splicing inhibition would make the host protein non-functional (Paulus, 2003; Liu and Yang, 2004). However, drug screening for the inhibition of intein splicing has only been carried out for bacterial pathogens, such as *M. tuberculosis.* The intein MtuRecA was inserted in the GFP (green fluorescent protein) coding sequence and its splicing efficiency was evaluated according to the fluorescence emission. More than 85 thousand compounds were tested. Some electrophilic compounds, such as cisplatin inhibited the intein MtuRecA splicing by blocking the intein’s first *N*-Spl cystein residue (Paulus, 2007; Zhang et al., 2010).

Three host proteins, considered essential for cell physiology, have been observed containing inteins in Saccharomycetales species: vacuolar ATPase (VMA), threonyl-tRNAsynthetase (ThrRS) and glutamate synthase (GLT1) (Poulter et al., 2007).

In order to update the distribution of these inteins in *Candida*, we carried out a search for inteins sequences in 81 sequenced genomes of 30 *Candida* species (Supplementary Table 1). Public Contigs deposited into MycoCosmosDB and NCBI WGS Databases were downloaded. By using GetORF program, all genomes were converted into six frames amino-acids sequences, for their ORFs deduction. Using those sequences, a local database was created, using NCBI Local BLAST+ (v.3.1.4) program in a UNIX system. Finally, sequences of VMA, ThrRS, GLT1 inteins were blasted to this database. This analysis revealed inteins in VMA, ThrRS and GLT1 proteins in *Candida* species, in which they have not been described before.

The VMA, ThrRS and GLT1 inteins are present in 14, 6, and 3 *Candida* species, respectively. The differences among *Candida* species concerning intein presence and/or size polymorphism (**Table 1**) can be easily accessed as molecular markers for species differentiation.

**Table 1:** Inteins encoded by *Candida* spp.

Host protein	Candida species	Intein nomenclature	Size(aa residues)	Essential aspartate residue^∗^	
				Block C	Block E
VMA1, vacuolar ATPase	*C. castellii^∗∗^*	CcaVMA	367	D	I
	*C. apicola^∗∗^*	CapVMA	389	T	K
	*C. glabrata*	CglVMA	415	S	Q
	*C. nivariensis^∗∗^*	CniVMA	424	S	K
	*C. bracarensis^∗∗^*	CbrVMA	420	S	K
	*C. homilentoma^∗∗^*	ChoVMA	491	D	D
	*C. sorboxylosa^∗∗^*	CsorVMA	374	T	V
	*C. tropicalis*	CtrVMA	471	I	A
	*C. sojae^∗∗^*	CsoVMA	501	D	N
	*C. metapsilosis*	CmeVMA	454	N	D
	*C. orthopsilosis*	CorVMA	530	D	D
	*C. intermedia^∗∗^*	CinVMA	455	T	D
	*C. maltosa^∗∗^*	CmaVMA	451	N	D
	*C. famata*	CfaVMA (DhaVMA)	395	N	D
ThrRS, threonyl-tRNA synthetase	*C. tropicalis*	CtrThrRS	345	N	S
	*C. sojae^∗∗^*	CsoThrRS	338	T	S
	*C. maltosa^∗∗^*	CmaThrRS	444	D	D
	*C. orthopsilosis*	CorThrRS-A	180	–	–
	*C. orthopsilosis*	CorThrRS-B	439	D	D
	*C. parapsilosis*	CpaThrRS	183	–	–
	*C. metapsilosis*	CmeThrRS	172	–	–
GLT1, glutamate synthase	*C. famata*	CfaGLT1	607	D	D
	*C. carpophila^∗∗^*	Ccar GLT1	557	D	D
	*C. guilliermondii*	Cgu GLT1	553	E	D

*^∗^Aspartic acids residues from block C and E considered essential for HE domain function. ^∗∗^Inteins found in new available *Candida* genomes.*

Some *Candida* species have more than one intein in their genome. The species *C. maltosa, C. metapsilosis* and *C. orthopsilosis* present both VMA and ThrRS inteins and *C. famata* presents both VMA and GLT1 inteins (**Table 1**). No inteins were found in *C. albicans, C. arabinofermentans, C. auris, C. boidinii, C. dubliniensis, C. succiphila, C. tanzawaensis, C. tenuis, C. infanticola, C. krusei, C. lusitaniae*, and *C. ciferri*.

Inteins are not exclusively found in clinically relevant *Candida* species, some of the intein containing species listed on **Table 1** are frequently isolated from environmental sources. For instance, *C. apicola* is found in wine and cachaça fermentation processes (Vega-Alvarado et al., 2015), *C. homilentoma* is commonly associated to insects (Yun et al., 2015), *C. sorboxylosa* was originally found in fruits and described as close related to *C. krusei* (Nakase, 1971) and *C. sojae* can be isolated from water-soluble substances of defatted soybean flakes (Nakase et al., 1994).

Some environmental species presenting inteins, listed on **Table 1**, have been rarely reported in invasive candidemia in human. This is the case of *C. intermedia*, isolated from soil, beer, grapes and also present on skin, throat, or animal feces (Ruan et al., 2010), as well as *C. famata* (*Debaryomyces hansenii*), which is commonly found in natural substrates and in various types of cheese and accounts for 0.08–0.5% of isolates recovered during invasive candidiasis. *C. famata* is sometimes misidentified as *C. guilliermondii*, which has a variety of environmental sources and is a common constituent of the normal human microbiota, being associated with only 1–2% of candidemias (Desnos-Ollivier et al., 2008). The species *C. castellii, C. nivariensis* and *C. bracarensis* belong to *Nakaseomyces* genus (Kurtzman, 2003), being closely related to *C. glabrata.* The high genetic proximity between *C. nivariensis* or *C. bracarensis* and *C. glabrata* may have caused their misidentification as *C. glabrata* (Alcoba-flórez et al., 2005; Correia et al., 2006; Bishop et al., 2008), so that their emergent character may have been due to recent advances in molecular epidemiology tools. The ecological niches of these species are still poorly understood. *C. nivariensis* has been isolated from flowering plants in Australia, indicating a possible environmental source for human infections (Lachance et al., 2001). Despite their environmental aspect, *C. nivariensis* and *C. bracarensis* show an expansion in EPA genes, a family of glycosylphosphatydylinositol (GPI)-anchored cell-wall protein, considered an important virulence factor for emergent pathogens, such as *C. glabrata* (Gabaldón et al., 2013).

### VMA Inteins

The first evidence of an intein arose from structural studies and expression analysis of the vacuolar ATPase gene and its encoded protein (VMA) in *S. cerevisiae* (Hirata et al., 1990). Since then, VMA inteins have been described in several species of Saccharomycetales order, including yeasts of biotechnological and medical interests, and seem to follow a model of invasion, fixation, degeneration, loss and reinvasion (Okuda et al., 2003; Burt and Koufopanou, 2004; Butler et al., 2006), leading to its sporadic distribution. For this reason two very close sister species might differ concerning the presence of the intein in the *VMA* gene. Most of the VMA inteins have a degenerated HE domain (from LAGLIDADG family). Some of the few active HE may also recognize allelic sites in other yeast species. Actually, the HE domain of the VMA intein (also called VDE) from *S. cariocanus* is more effective in cutting the *VMA* recognition site of *S. cerevisiae* than its own *VMA* (Posey et al., 2004), which would allow horizontal transfer. Notwithstanding, once the intein is acquired, it tends to be transmitted vertically, reflecting the group phylogeny (Butler et al., 2006; Goodwin et al., 2006). Comparing the VMA intein patterns in related *Saccharomyces* species, it was also possible to document hybridization process, such as in a diploid strain of *S. carslbergensis* that contain two distinct alleles, one with the VMA intein, supposedly received from *S. cerevisiae*, and the other with only the intein-less *VMA* sequence, probably received from *S. pastorianus* (Okuda et al., 2003).

The amino acid sequence alignment of VMA inteins in *Candida* species shows large degeneration process in the HE domain, mainly in those species lacking both aspartate residues, one in block C and the other in E (Supplementary Figure S1).

**FIGURE 2:**
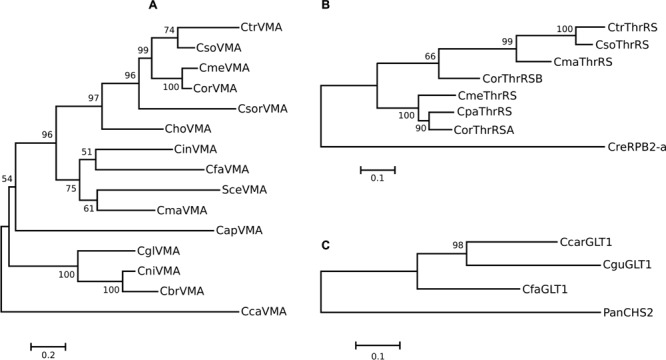
**Molecular Phylogenetic analysis by Maximum Likelihood method conducted in Mega v.6.0 (Tamura et al., 2013) for the amino acids aligned sequences of the inteins VMA, ThrRS and GLT1 from *Candida* and their close relative inteins.** The trees with the highest log likelihood are shown. The trees are drawn to scale, with branch lengths measured in the number of substitutions per site. All sites containing alignment gaps were removed (Complete deletion). **(A)** VMA intein phylogeny inferred based on the Le_Gascuel_2008 model (Le and Gascuel, 2008). A discrete Gamma distribution was used to model evolutionary rate differences among sites [5 categories (+*G*, parameter = 2.0642)] and the rate variation model allowed for some sites to be evolutionarily invariable ([+*I*], 8.7480% sites). **(B)** ThrRS intein phylogeny inferred based on the Whelan And Goldman model (Whelan and Goldman, 2001). The rate variation model allowed for some sites to be evolutionarily invariable. **(C)** GLT1 intein phylogeny inferred based on the Whelan And Goldman model (Whelan and Goldman, 2001). The rate variation model allowed for some sites to be evolutionarily invariable.

The phylogenetic relationships among these elements revealed a non-vertically inheritance pattern, since the intein from *C. maltosa* was clustered apart from *C. tropicalis, C. metapsilosis* and *C. orthopsilosis* inteins (**Figure 2A**). According to a combined maximum likelihood analysis of six genes (*ACT1, EF2, RPB1, RPB2, 18S* rDNA, and *26S* rDNA) (Diezmann et al., 2004) these four species belong to a unique clade, named clade 1. Besides, although *C. glabrata, C. castellii, C. nivariensis* and *C. bracarensis* share a common ancestor with *S. cerevisiae*, belonging to clade 3 (Diezmann et al., 2004), their inteins were not clustered together in our analysis. Actually, the intein SceVMA showed to be closer to CmaVMA, than to the inteins of other species from clade 3. These observations corroborate the hypothesis of VDE adaptation for horizontal transfer. This hypothesis is supported by the high conservation of its 31 bp long recognition site and also by the incongruence between host and inteins phylogenies. This horizontal transfer seems to occur preferentially between closely related species, probably by eventual hybridization events (Goddard and Burt, 1999; Koufopanou et al., 2002).

Horizontal transfer has also been proposed as an explanation to the peculiar distribution of other inteins. For instance, the PRP8 intein, which occurs, sporadically, in many ascomycetes is also found in only four basidiomycetes from Tremellales order (*Cryptococcus neoformans, Cryptococcus gattii, Cryptococcus laurentii* and *Cryptococcus bacillisporus*) (Butler and Poulter, 2005; Butler et al., 2006). It was speculated that a co-phagocytosis event by a metazoan macrophage could be a possible scenario for PRP8 intein transfer from an ascomycete to a basidiomycete (Poulter et al., 2007).

It seems plausible that, for sharing part of their ecological niches, such as metazoan tissues, as well as some environmental sources, and being closely related, *Candida* species could hybridize creating suitable conditions for HE invasion in new and empty alleles, carrying the VMA intein. In fact, there are some researches that point out the occurrence of hybridization events in *Candida*. For instance, hybrid lineages between the two subspecies of *C. orthopsilosis* have been described in distant continents (Pryszcz et al., 2014). The same authors also found genomic evidence that some *C. metapsilosis* strains worldwide distributed are heterozygous hybrids resulting from the same past hybridization event involving two non-pathogenic parental lineages. This observation corroborates the idea of hybridization as a source for the emergence of virulence attributes (Pryszcz et al., 2015).

The vacuolar H+-ATPase is a fundamental and therefore highly conserved enzyme in almost every eukaryotic cell. It functions as ATP-dependent proton pumps energizing various organelles and membranes, making numerous secondary transport processes possible. Yeast genetics researches identified the properties of individual subunits of V-ATPase and discovered the factors involved in its biogenesis and assembly. Null mutations in genes encoding V-ATPase subunits of *S. cerevisiae* result in a phenotype that is unable to grow at high pH and is sensitive to high and low metal-ion concentrations (Nelson et al., 2000).

The VMA intein is inserted in a P-loop containing the Nucleoside Triphosphate Hydrolase domain of VMA protein, the most prevalent domain of the several distinct nucleotide-binding protein folds. The most common reaction catalyzed by enzymes of the P-loop NTPase fold is the hydrolysis of the beta-gamma phosphate bond of a bound nucleoside triphosphate (NTP). The energy from NTP hydrolysis is typically utilized to induce conformational changes in other molecules, which constitutes the basis of the biological functions of most P-loop NTPases (Leipe et al., 2004).

The VMA intein is sporadically distributed among *Candida* species, with very close related species differing in its presence. For instance, this intein is absent in *C. parapsilosis*, while it is present, in different sizes, in *C. orthopsilosis* and *C. metapsilosis* (as described on **Table 1**), although *C. orthopsilosis* and *C. parapsilosis* share a most recent common ancestor, being closer to each other than either of them to *C. metapsilosis* (Pryszcz et al., 2015). It is possible that the clonal nature of *C. parapsilosis* lineage (Tavanti et al., 2005), in contrast to the possible mating occurrence in *C. orthopsilosis* and *C. metapsilosis* (Sai et al., 2011; Pryszcz et al., 2015), could have contributed to the loss of VMA intein. On the other hand, the sexual reproductive mode, as well as the occurrence of hybridization events, combined to a certain adaptation for lateral transfer of VDE, would prevent the intein loss in *C. orthopsilosis* and *C. metapsilosis.* The presence/absence and size polymorphisms make the VMA intein an easy strategy to differentiate these cryptic species (Prandini et al., 2013), constituting the most practical DNA-based method proposed so far for the correct identification of the species from the complex, since it does not requires PCR digestion or sequencing. However, additional retrospective analysis of more isolates known to belong to *C. parapsilosis* complex should be carried out in order to better explore the VMA intein as a phylogenetic tool.

Zhang and Rao (2010) discussed the contributions of V-ATPase function to pathogenicity and reviewed the functional link between V-ATPase and the lipid components of the membrane, showing that ergosterol removing or inhibition (performed by azoles, morpholines and allylamines) alters the V-ATPase conformation. Also, *erg* mutants showed the same phenotype of *vma* mutants, which is the inability to grow in alkaline medium. Besides, the authors also pointed out that fluconazole treatment, as well as *ERG3* deletion or *vma7-/-* mutation cause inhibition of filamentation, which, for *Candida* pathogenic species, is an important virulence treat for tissue invasion (Lo et al., 1997; Saville et al., 2006; Bastidas and Heitman, 2009). Furthermore, *C. albicans vma7-/-* mutant cells are eliminated by macrophages and fail to colonize epithelial cells. These observations show that azole drugs may have an effect on V-ATPase function, disrupting the pH homeostasis in fungal pathogens (Zhang and Rao, 2010). Despite no data is available for *vma* mutants in non-albicans *Candida* species, the conservative aspect of the VMA protein makes plausible the assumption that this protein is actually essential for the survival of all yeast species, mainly in alkaline medium, and it may also play an important role for fungal maintenance during infection in other *Candida* species. For this reason, we suppose this data reinforce the importance of the intein in V-ATPase protein as a potential drug target for the inhibition of the normal function of this protein in those *Candida* species that present this genetic element.

It is interesting to note that the most prevalent *Candida* species in hospital infections is *C. albicans*, which does not have any intein, while non-albicans *Candida* spp. predominated in samples collected from environment (Ferreira et al., 2013). If we consider that some physiological conditions may decrease the intein splicing efficiency, the existence of an intein in an important protein can modulate its post-translational expression. Regarding the importance of the V-ATPase for cell homeostasis and even for filamentation, it seems reasonable that the loss of this intein in *C. albicans* lineage might have contributed, together with many virulence factors, for its maintenance in vertebrate tissue as a member of the normal microbiota and eventual pathogen. The absence of the intein in VMA protein could have contributed, for example, for the highly efficient filamentation of *C. albicans* when compared to other *Candida* species, that have the VMA intein, such as *C. glabrata* and *C. tropicalis*, whose pathogenicity might be associated with many other virulence factors rather than to filamentation capacity (Sudbery, 2011). Among the non-albicans *Candida* spp., whose incidence is becoming more expressive, are the species *C. parapsilosis* and *C. krusei*, which also lack the VMA intein. In these species, the *VMA* gene expression is not under post-translational regulation controlled by intein splicing, so that it could be more efficiently expressed in a larger variety of physiological conditions when compared to the intein containing species. However, no experiment has been conducted to assess the splicing of the VMA intein in *Candida* species in different physiological and stressing conditions.

### ThrRS Intein

The ThrRS intein is inserted in the threonyl-tRNAsynthetase gene (*THRRS*) which encodes an aminoacyl-tRNAsynthetase (aaRS), responsible for engaging the amino acid threonine with the corresponding tRNA (anticodon). The ThrRS intein is located in the Class II tRNA aaRS catalytic core domain. Class II amino acyl-tRNA synthetases (aaRSs) share a common fold and generally attach an amino acid to the 3′ OH of the tRNA ribose. This domain is primarily responsible for ATP-dependent formation of the enzyme bound aminoacyl-adenylate (O’Donoghue and Luthey-Schulten, 2003).

The ThrRS intein has already been described as full-length intein in *C. tropicalis* and as mini-intein in *C. parapsilosis*, as well as in *C. orthopsilosis* (CorThrRS-A) and *C. metapsilosis.* Nevertheless, some isolates of *C. orthopsilosis* present a full-length intein (CorThrRS-B) in the same insertion site. This was the first report of two types of intein in the same insertion site in the same species (Prandini et al., 2013), though in distinct strains. The finding of both inteins (CorThrRS-A and CorThrRS-B in the same *C. orthopsilosis* strain is also possible, since they are diploids. Here we also described a full-length intein in *C. maltosa* (CmaThrRS), which, like the CorThrRS-B, presents both aspartic acids residues (D), and in *C. sojae* (CsoThrRS), whose aspartic acid residues were replaced by the amino acids T and S (**Table 1**; Supplementary Figure S2).

Phylogeny of the ThrRS inteins clearly distinguished the *Candida* species from the *C. parapsilosis* complex (**Figure 2B**), but it does not corroborate the species phylogeny initially proposed, in which *C. metapsilosis* and *C. orthopsilosis* share a most recent common ancestor and are the sister clade of *C. parapsilosis* (Tavanti et al., 2005; Wolfe et al., 2012). However, a more recent phylogenetic analysis using 396 conserved, as well as a super-tree derived from the whole phylome using a gene tree parsimony approach, supported a basal position of *C. metapsilosis* to the exclusion of *C. orthopsilosis* and *C. parapsilosis* (Pryszcz et al., 2015).

The splicing domain of the intein CorThrRS-B (a full-length intein) does not group with the other intein of *C. orthopsilosis*, the CorThrRS-A (a mini intein), since it is more closely related to ThrRS inteins from *C. tropicalis, C. maltosa* and *C. sojae* (**Figure 2B**). This might reflect the occurrence of independent intein invasions. The ancestor of *C. orthopsilosis* species might have its *THRRS* gene invaded by an intein (ThrRS-A), which, following the homing cycle “rules,” might have been fixed in most of population, leading to its HE degeneration (explaining its current mini-intein structure). Since the homing endonuclease is no longer functional, empty sites could have arisen being occupied again by another intein, the ThrRS-B, which also invaded the *THRRS* gene from *C. tropicalis, C. sojae*, and *C. maltosa.*

**FIGURE 3:**
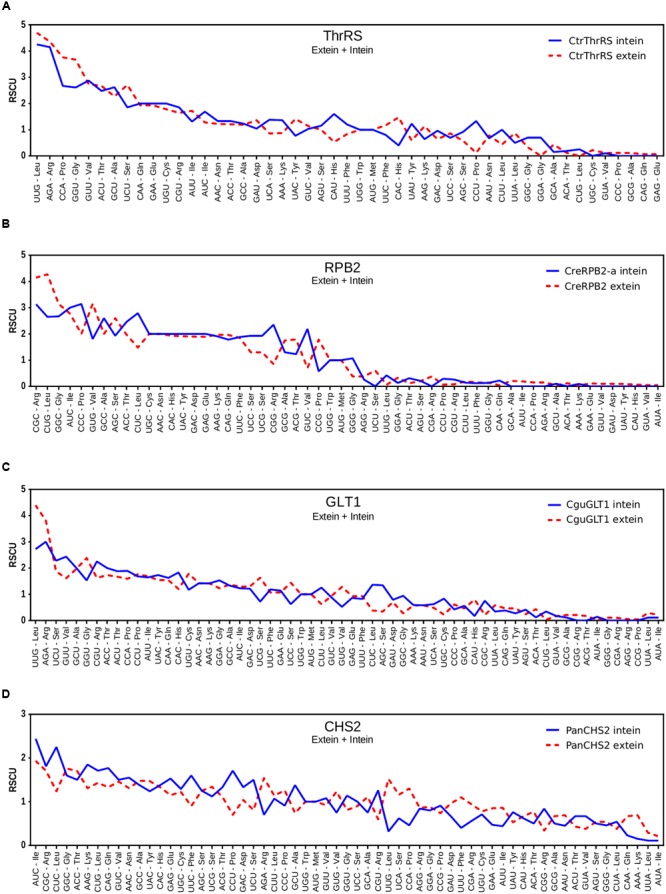
**Graphic view of RSCU values for codon usage in ThrRS, GLT1, RBP2 and CHS2 inteins and their respective exteins.** Comparison between: **(A)** RSCU values for CtrThrRS intein and *THRRS* gene of *C. tropicalis* without intein; **(B)** RSCU values for CreRBP2-a intein and *RPB2* gene of *C. reinhardtii* without intein; **(C)** RSCU values for CguGLT1 intein and *GLT1* gene of *C. tropicalis* without intein and **(D)** RSCU values for PanCHS2 intein and *CHS2* gene of *P. anserina* without intein.

The ThrRS intein has 29.33% of similarity with one of the four inteins located in the RNA polymerase II of *Chlamydomonas reinhardtii* (the CreRPB2-a intein), a green unicellular algae. Indeed, the RPB2-a intein was discovered because of its similarity to the threonyl-tRNA synthetase intein from *C. tropicalis*, which suggest the occurrence of horizontal transfer (Goodwin et al., 2006; Poulter et al., 2007). This horizontal transfer, if recent, can be evidenced by the sequence similarity between the exteins and between the inteins and also by differences in codon usage between intein and extein, as demonstrated for the DnaB intein and its extein in *Rhodothermus marinus* (Liu and Hu, 1997). If two homologous inteins are not related through recent lateral transfer, the sequence divergence between them is larger than between their extein sequences, in a similar way that happens to introns, whose sequences usually diverge faster than exon sequences. Accordingly, in the well-known case of lateral transfer of DnaB intein from *Synechocystis* sp. to *R. marinus*, the intein aminoacid sequences share a 54% sequence identity that is noticeably higher than the 37% sequence identity shared by the DnaB extein sequences (Liu and Hu, 1997). In the case of the CtrThrRS and CreRPB2 inteins: their identity is 29.33%, which is higher than the 17.77% sequence identity shared by the extein sequences. But, of course, this low identity is already expected since these exteins are not homologous genes. However, no pronounced deviation in relative synonymous codon usage (RSCU) (Sharp et al., 1986) is observed between the ThrRS intein and its extein or between the RPB2-a intein and its extein, suggesting that the possible lateral transfer was not a recent event (**Figures 3A,B**). The same is not observed between both inteins and also between both exteins, which present a great codon usage deviation (Supplementary Figures S4A,B).

The aaRSs are considered important therapeutic target for antibiotics, antifungals and antiprotozoal drugs. Most inhibitors of aaRSs act by competitive binding at the active site where normally the cognate amino acid would bind (Vondenhoff and Van Aerschot, 2011; Kalidas et al., 2014). Most of these compounds are not commercial and few have reached the stage of clinical development. Icofungipen, for example is an antifungal that inhibits IleRS, presenting satisfactory clinical efficacy and safety, although low mycological eradication rates were observed in HIV-positive patients (Ochsner et al., 2007). Cispentacin, a cyclic β-amino acid that has been isolated from *Bacillus cereus* and *Streptomyces setonii* inhibits IleRS and proved to be effective against *C. albicans* infection in mice (Konishi et al., 1989; Ochsner et al., 2007).

The presence of an intein in the ThrRS protein would represent an additional approach for its inhibition, as well as it would assure a specific and therefore safer antifungal mechanism, because, despite the functional evolutionary convergence, as all the aaRS carry out the same basic biochemical function (O’Donoghue and Luthey-Schulten, 2003), only the ThrRS protein of the fungal pathogen has an intervening intein.

### GLT1 Intein

The *GLT1* gene codifies an oligomeric enzyme named glutamate synthase (GOGAT), which is composed by three identical subunits. This enzyme, together with glutamine synthase, encoded by *GLN1* gene, is involved in one of the three pathways for the synthesis of glutamate, in yeast cells. GOGAT catalyzes the reductive synthesis of L-glutamate from 2-oxoglutarate and L-glutamine via intramolecular channeling of ammonia. It is a multifunctional enzyme that functions through three distinct active centers, carrying out L-glutamine hydrolysis, conversion of 2-oxoglutarate into L-glutamate and electron uptake from an electron donor (Filetici et al., 1996).

In this review we described, for the first time the GLT1 intein in *C. carpophila*, a distinct species that is closely related to both *P. guilliermondii* and *C. fermentati*. Before being described as a different species, *C. carpophila*, previously named as *C. guilliermondii* var. *carpophila*, was considered as a member of a genetically heterogeneous complex comprising several phenotypically indistinguishable taxa inside *C. guilliermondii* (Vaughan-Martini et al., 2005).

Both CfaGLT1 and CcarGLT1 inteins present the two aspartic acid residues, known to be essential for HE function, while CguGLT1 presented a substitution to E residue in the first aspartate (**Table 1**; Supplementary Figure S3). Phylogenetic analysis previously indicated that these inteins were very closely related to a non-allelic intein, the PanCHS2 (the intein in chitin synthase 2, from *Podospora anserina*) (Poulter et al., 2007). Similarly to that observed for the ThrRS intein, the RSCU between the GLT1 intein and its extein is concordant for most codons, also indicating that the possible lateral transfer of this intein from CHS2 to GLT1 is ancient (**Figures 3C,D**). The same is not observed between both inteins and also between both exteins, which present a great codon usage deviation (Supplementary Figures S4C,D).

As we can infer from **Figure 2C**, the GLT1 intein probably invaded the *GLT1* gene before the divergence of *Candida* species from the clade 2 proposed by Diezmann et al. (2004), which includes *C. guilliermondii, C. intermedia, C. famata* and *C. zeylanoides.* Evidently this intein was lost in *C. intermedia* and *C. zeylanoides*, probably due to HE degeneration and genetic drift.

According to KEEG Orthology (entry: YDL171C), the host protein for this intein, the GOGAT protein, is involved in cryptic pathways such as (i) alanine, aspartate and glutamate metabolism; (ii) nitrogen metabolism; (iii) biosynthesis of secondary metabolites; (iv) biosynthesis of antibiotics; and (v) biosynthesis of amino-acids. Connecting all these pathways, there is high nitrogen dependency. Nitrogen is a very important component, which is present in the chemical structure of almost every single molecule in cell’s constituents. Yeasts cannot make their own nitrogen by taking it from the air, so it is necessary external sources of this macronutrient to keep it constantly available. So, the cell needs to adapt their metabolism to obtain or catabolize the available nitrogen sources, such as ammonia, glutamine, asparagine, glutamate, and proteins.

As the life style of *Candida* ssp. ranges from environmental to commensal and/or parasitic, the nutrient availability varies as well. During infection, *Candida* ssp. needs to obtain nitrogen from a broad range of different sources, which may change dramatically depending on the anatomical site of infection. The changing from commensal to pathogenic growth depends on a differential gene expression, which allows the establishment of infection (Ramachandra et al., 2014).

*Candida* spp. are constantly exposed to stressful agents, such as nitrogen deprivation in their microenvironment changing. Also, it is known that nitrogen source utilization modulates some morphological and physiological changes, sexual and asexual sporulation and virulence factors expression (Marzluf, 1997; Biswas et al., 2007). Therefore, the presence of the intein in this protein, by a post-translational expression modulation, could allow fine adjustments in nitrogen pathway during infection.

GLT1 expression is highly modulated in *S. cerevisiae*, being repressed in the presence of glutamate rich nitrogen sources, which suggests that GOGAT may have an important role in glutamate biosynthesis under conditions where this amino acid becomes limiting. It seems that GOGAT constitute an ancillary pathway, supplying low but continuous glutamate production, even in the presence of other glutamate pathways, suggesting that a high intracellular glutamate concentration may be needed for optimal growth, mainly under conditions in which carbon and nitrogen are limiting (Valenzuela et al., 1998). *GLT1* knockout evaluation showed that GOGAT does not significantly influence cellular physiology, corroborating its auxiliary characteristic in glutamate synthesis (Brambilla et al., 2016), though GOGAT non-expression yeasts accumulated more ROS, when treated with hydrogen peroxide.

The use of different glutamate pathways in *Candida* was compared to Saccharomyces (Holmes et al., 1989). The authors compared the NADP+-dependent GDH and GOGAT pathways activities in four *Candida* species (*C. albicans, Candida pseudotropicalis, C. parapsilosis* and *C. tropicalis*) and *S. cerevisiae*. The relative contribution of GOGAT in *S. cerevisiae* is around 1.6%, while among *Candida* species it ranges from 13 to 70%, being most expressive in *C. albicans.* This observation may indicate that, in *Candida*, GOGAT pathway may have a greater impact in nitrogen metabolism than in *S. cerevisiae*, so a post-translational regulatory role of the intein in this gene would be noteworthy in *Candida.* Thus, if the relative contribution of GOGAT is as expressive in non-albicans *Candida* species containing GLT1 intein as it is in *C. albicans*, the GLT1 intein could also be considered an additional drug target.

## Conclusion

In this review, the list of intein containing *Candida* species was updated, as well as their sequence features and phylogenetic relationships. The horizontally transferred nature of VMA inteins was corroborated by our phylogeny. The possible lateral transfer of inteins from *C. reinhardtii* (*RPB2* gene) and *P. anserina* (*CHS2* gene) to *THRRS* and *GLT1* genes, respectively, of *Candida* species, might be very ancient, since no significant deviation of RSCU was observed between these inteins and their respective exteins. Besides the peculiar evolutionary history of the inteins, their presence or absence, as well as their polymorphic sizes, should also be explored as molecular markers for species or cryptic species recognition, assisting the diagnosis and the therapeutic choices, since different species diverge in their clinical and antifungal susceptibility aspects.

The recent discussions about whether inteins are parasitic genetic elements or post-translational expression modulators gives rise to important questions: Can the splicing efficiency of an intein be altered under different life-style conditions of opportunistic or pathogenic fungal species? Can inteins regulate gene expression in different ways during infection? Here we reviewed the importance of the *VMA, ThrRS* and *GLT1* genes in the yeast cell physiology. Besides acidifying vacuoles, the VMA protein can also be important for fungal filamentation, an important virulence feature in *C. albicans*. Can the presence of an intein in the VMA protein modulate its function in the intein-containing *Candida* species, interfering in their filamentation capacity? The ThrRS protein is essential for protein synthesis in cell; the splicing inefficiency of its intein would prevent cell proliferation and maintenance. This protein is inhibited by some drugs and the presence of an intein in some species would add another way to target it, by inhibiting its intein splicing. The GOGAT protein, coding by *GLT1* gene, on the other hand, is not considered so essential because it is part of one out of three possible pathways to synthetize glutamate, however, since this pathway seems to be largely used in some *Candida* species, the GLT1 intein could also constitute an important therapeutic target for those non-albicans *Candida* species that contain it.

In order to address the aforementioned questions many experimental researches must be done, clarifying the actual role of inteins in fungal pathogens and possibly opening new prospects for antifungal drugs researches. The use of inteins as a new drug target is, clearly, limited to the species in which they are present in at least one protein, while their absence/presence should also be explored as a molecular marker. The merit of this discussion can be extended for other pathogenic fungi, besides *Candida* spp., that contain inteins in housekeeping genes, and may open a new study field in medical mycology concerning invasive genetic elements as tools for drug screening and diagnosis.

## Author Contributions

JF, TP, and RT conceived, designed, did the literature review, provided and wrote the manuscript. JF carried out the search for inteins in *Candida* genomes and the RSCU analysis. TA, MC, and JG reviewed the taxonomy and epidemiology of *Candida* species. TA, MC, JG, and EB assisted in the preparation, final review, and co-wrote the manuscript.

## Conflict of Interest Statement

The authors declare that the research was conducted in the absence of any commercial or financial relationships that could be construed as a potential conflict of interest.

## References

[B1] Alcoba-flórez J. Méndez-álvarez S. Guarro J. Pérez-roth E. Arévalo P. Cano J. (2005). Phenotypic and molecular characterization of *Candida nivariensis* sp. nov., a possible new opportunistic fungus. *J. Clin. Microbiol.* 43 4107–4111. 10.1128/JCM.43.8.4107 16081957 PMC1233986

[B2] Bailey D. Diamandis E. P. Greub G. Poutanen S. M. Christensen J. J. Kostrzew M. (2013). Use of MALDI-TOF for diagnosis of microbial infections. *Clin. Chem.* 59 1435–1441. 10.1373/clinchem.2013.204644 23695295

[B3] Bassetti M. Merelli M. Ansaldi F. De Florentiis D. Sartor A. Scarparo C. (2015). Clinical and therapeutic aspects of candidemia: a five year single centre study. *PLoS ONE* 10:e0127534 10.1371/journal.pone.0127534 PMC4444310 26010361

[B4] Bastidas R. J. Heitman J. (2009). Trimorphic stepping stones pave the way to fungal virulence. *Proc. Natl. Acad. Sci. U.S.A.* 106 351–352. 10.1073/pnas.0811994106 19129500 PMC2626706

[B5] Belfort M. Bonocora R. P. (2014). Homing endonucleases: from genetic anomalies to programmable genomic clippers. *Methods Mol. Biol.* 1123 1–26. 10.1007/978-1-62703-968-0_1 24510256 PMC4436680

[B6] Bishop J. A. Chase N. Magill S. S. Kurtzman C. P. Fiandaca M. J. Merz W. G. (2008). *Candida* bracarensis detected among isolates of *Candida glabrata* by peptide nucleic acid fluorescence in situ hybridization: susceptibility data and documentation of presumed infection. *J. Clin. Microbiol.* 46 443–446. 10.1128/JCM.01986-07 18077641 PMC2238114

[B7] Biswas S. Van Dijck P. Datta A. (2007). Environmental sensing and signal transduction pathways regulating morphopathogenic determinants of *Candida albicans*. *Microbiol. Mol. Biol. Rev.* 71 348–376. 10.1128/MMBR.00009-06 17554048 PMC1899878

[B8] Borman A. M. Linton C. J. Oliver D. Palmer M. D. Szekely A. Odds F. C. (2009). Pyrosequencing analysis of 20 nucleotides of internal transcribed spacer 2 discriminates *Candida parapsilosis, Candida metapsilosis*, and *Candida orthopsilosis*. *J. Clin. Microbiol.* 47 2307–2310. 10.1128/JCM.00240-09 19403763 PMC2708508

[B9] Brambilla M. Adamo G. M. Frascotti G. Porro D. Branduardi P. (2016). Physiological effects of GLT1 modulation in *Saccharomyces cerevisiae* strains growing on different nitrogen sources. *J. Microbiol. Biotechnol.* 26 326–336. 10.4014/jmb.1508.08002 26528537

[B10] Burt A. Koufopanou V. (2004). Homing endonuclease genes: the rise and fall and rise again of a selfish element. *Curr. Opin. Genet. Dev.* 14 609–615. 10.1016/j.gde.2004.09.010 15531154

[B11] Butler M. I. Goodwin T. J. D. Poulter R. T. M. (2001). A nuclear-encoded intein in the fungal pathogen *Cryptococcus neoformans*. *Yeast* 18 1365–1370. 10.1002/yea.781 11746598

[B12] Butler M. I. Gray J. Goodwin T. J. D. Poulter R. T. M. (2006). The distribution and evolutionary history of the PRP8 intein. *BMC Evol. Biol.* 6:42 10.1186/1471-2148-6-42 PMC1508164 16737526

[B13] Butler M. I. Poulter R. T. M. (2005). The PRP8 inteins in *Cryptococcus* are a source of phylogenetic and epidemiological information. *Fungal Genet. Biol.* 42 452–463. 10.1016/j.fgb.2005.01.011 15809009

[B14] Chong S. Shao Y. Paulus H. Benner J. Perler F. B. Xu M. (1996). Protein splicing involving the *Saccharomyces cerevisiae* VMA intein. *J. Biol. Chem.* 271 22159–22168. 10.1074/jbc.271.36.22159 8703028

[B15] Cleveland A. A. Farley M. M. Harrison L. H. Stein B. Hollick R. Lockhart S. R. (2012). Changes in incidence and antifungal drug resistance in candidemia: results from population-based laboratory surveillance in Atlanta and Baltimore, 2008-2011. *Clin. Infect. Dis.* 55 1352–1361. 10.1093/cid/cis697 22893576 PMC4698872

[B16] Cleveland A. A. Harrison L. H. Farley M. M. Hollick R. Stein B. Chiller T. M. (2015). Declining incidence of candidemia and the shifting epidemiology of *Candida* resistance in two US metropolitan areas, 2008–2013: results from population-based surveillance. *PLoS ONE* 10:e0120452 10.1371/journal.pone.0120452 PMC4378850 25822249

[B17] Colombo A. L. Guimarães T. Camargo L. F. A. Richtmann R. de Queiroz-Telles F. Salles M. J. C. (2013). Brazilian guidelines for the management of candidiasis – a joint meeting report of three medical societies: Sociedade Brasileira de Infectologia, Sociedade Paulista de Infectologia and Sociedade Brasileira de Medicina Tropical. *Braz. J. Infect. Dis.* 17 283–312. 10.1016/j.bjid.2013.02.001 23693017 PMC9427385

[B18] Correia A. Sampaio P. James S. Pais C. (2006). *Candida* bracarensis sp. nov., a novel anamorphic yeast species phenotypically similar to *Candida glabrata*. *Int. J. Syst. Evol. Microbiol.* 56 313–317. 10.1099/ijs.0.64076-0 16403904

[B19] De Carolis E. Vella A. Vaccaro L. Torelli R. Posteraro P. Ricciardi W. (2014). Development and validation of an in-house database for matrix assisted laser desorption ionization–time of flight mass spectrometry-based yeast Identification using a fast protein extraction procedure. *J. Clin. Microbiol.* 52 1453–1458. 10.1128/JCM.03355-13 24554755 PMC3993681

[B20] Desnos-Ollivier M. Ragon M. Robert V. Raoux D. Gantier J. C. Dromer F. (2008). *Debaryomyces hansenii* (*Candida* famata), a rare human fungal pathogen often misidentified as *Pichia guilliermondii* (*Candida guilliermondii*). *J. Clin. Microbiol.* 46 3237–3242. 10.1128/JCM.01451-08 18701668 PMC2566122

[B21] Diezmann S. Cox C. J. Scho G. Vilgalys R. J. Mitchell T. G. (2004). Phylogeny and evolution of medical species of *Candida* and related taxa: a multigenic analysis. *J. Clin. Microbiol.* 42 5624–5635. 10.1128/JCM.42.12.5624 15583292 PMC535224

[B22] Fahami S. Kordbacheh P. Moazeni M. Mahmoodi M. Mirhendi H. (2010). Species identification and strain typing of *Candida* isolates by PCR-RFLP and RAPD-PCR analysis for determining the probable sources of nosocomial infections. *Iranian Red Crescent Med. J.* 12 539–547.

[B23] Ferreira A. V. Prado C. G. Carvalho R. R. Dias K. S. T. Dias A. L. T. (2013). *Candida albicans* and Non-*C. albicans Candida* species: comparison of biofilm production and metabolic activity in biofilms, and putative virulence properties of isolates from hospital environments and infections. *Mycopathologia* 175 265–272. 10.1007/s11046-013-9638-z 23532754

[B24] Filetici P. Martegani M. P. Valenzuela L. González A. Ballario P. (1996). Sequence of the GLT1 gene from *Saccharomyces cerevisiae* reveals the domain structure of yeast glutamate synthase. *Yeast* 12 1359–1366. 10.1002/(SICI)1097-0061(199610)12:13<1359::AID-YEA3>3.0.CO;2-5 8923741

[B25] Gabaldón T. Martin T. Marcet-Houben M. Durrens P. Bolotin-Fukuhara M. Lespinet O. (2013). Comparative genomics of emerging pathogens in the *Candida glabrata* clade. *BMC Genomics* 14:623 10.1186/1471-2164-14-623 PMC3847288 24034898

[B26] Gacser A. Schafer W. Nosanchuk J. S. Salomon S. Nosanchuk J. D. (2007). Virulence of *Candida parapsilosis, Candida orthopsilosis* and *Candida metapsilosis* in reconstituted human tissue models. *Fungal Genet. Biol.* 44 1336–1341. 10.1016/j.fgb.2007.02.002 17391997

[B27] Ghosh A. K. Paul S. Sood P. Rudramurthy S. M. Rajbanshi A. Jillwin T. J. (2015). Matrix-assisted laser desorption ionization time-of-fight mass spectrometry for the rapid identification of yeasts causing bloodstream infections. *Clin. Microbiol. Infect.* 21 372–378. 10.1016/j.cmi.2014.11.009 25658527

[B28] Gimble F. S. Thorner J. (1992). Homing of a DNA endonuclease gene by meiotic gene conversion in *Saccharomyces cerevisiae*. *Nature* 357 301–306. 10.1038/357301a0 1534148

[B29] Goddard M. R. Burt A. (1999). Recurrent invasion and extinction of a selfish gene. *Proc. Natl. Acad. Sci. U.S.A.* 96 13880–13885. 10.1073/pnas.96.24.13880 10570167 PMC24159

[B30] Gogarten J. P. Hilario E. (2006). Inteins, introns, and homing endonucleases: recent revelations about the life cycle of parasitic genetic elements. *BMC Evol. Biol.* 6:94 10.1186/1471-2148-6-94 PMC1654191 17101053

[B31] Goodwin T. J. D. Butler M. I. Poulter R. T. M. (2006). Multiple, non-allelic, intein-coding sequences in eukaryotic RNA polymerase genes. *BMC Biol.* 4:38 10.1186/1741-7007-4-38 PMC1635734 17069655

[B32] Gurbuz M. Kaleli I. (2010). Molecular analysis of *Candida albicans* isolates from clinical specimens. *Mycopathologia* 169 261–267. 10.1007/s11046-009-9263-z 20012366

[B33] Hays C. Duhamel C. Cattoir V. Bonhomme J. (2011). Rapid and accurate identification of species belonging to the *Candida parapsilosis* complex by real-time PCR and melting curve analysis. *J. Med. Microbiol.* 60 477–480. 10.1099/jmm.0.026633-0 21183600

[B34] Higashi C. M. Takashina F. H. Zendrini D. Stipp-abe A. T. Vespero E. C. Mariuza R. (2015). Comparison of Vitek-2 automated identification system and PCR-ITS for species characterization of clinical isolates. *Semina* 36 233–242. 10.5433/1679-0367.2015v36n1Suplp233

[B35] Hirata R. Ohsumi Y. Nakano A. Kawasaki H. Suzuki K. Anraku Y. (1990). Molecular structure of a gene, VMA1, encoding the catalytic subunit of H(+)-translocating adenosine triphosphatase from vacuolar membranes of *Saccharomyces cerevisiae*. *J. Biol. Chem.* 265 6726–6733. 2139027

[B36] Holmes A. R. Collings A. Farnden K. J. Shepherd M. G. (1989). Ammonium assimilation by *Candida albicans* and other yeasts: evidence for activity of glutamate synthase. *J. Gen. Microbiol.* 135 1423–1430. 2575653 10.1099/00221287-135-6-1423

[B37] Kalidas S. Cestari I. Monnerat S. Li Q. Regmi S. Hasle N. (2014). Genetic validation of aminoacyl-tRNA synthetases as drug targets in *Trypanosoma brucei*. *Eukaryot. Cell* 13 504–516. 10.1128/EC.00017-14 24562907 PMC4000095

[B38] Karahan Z. Güriz H. Agirbasli H. Balaban N. Gocmen J. S. Aysev D. (2004). Genotype distribution of *Candida albicans* isolates by 25S intron analysis with regard to invasiveness. *Mycoses* 47 465–469. 10.1111/j.1439-0507.2004.01022.x 15601450

[B39] Konishi M. Nishio M. Saitoh K. Miyaki T. Oki T. Kawaguchi H. (1989). Cispentacin, A new antifungal antibiotic I. Production, isolation, physico-chemical properties and structure. *J. Antibiot. (Tokyo)* 42 1749–1755. 10.7164/antibiotics.42.1749 2516082

[B40] Koufopanou V. Burt A. (2005). Degeneration and domestication of a selfish gene in yeast: molecular evolution versus site-directed mutagenesis. *Mol. Biol. Evol.* 22 1535–1538. 10.1093/molbev/msi149 15843599

[B41] Koufopanou V. Goddard M. R. Burt A. (2002). Adaptation for horizontal transfer in a homing endonuclease. *Mol. Biol. Evol.* 19 239–246. 10.1093/oxfordjournals.molbev.a004077 11861883

[B42] Kurtzman C. P. (2003). Phylogenetic circumscription of *Saccharomyces, Kluyveromyces* and other members of the Saccharomycetaceae, and the proposal of the new genera *Lachancea, Nakaseomyces, Naumovia, Vanderwaltozyma* and *Zygotorulaspora*. *FEMS Yeast Res.* 4 233–245. 10.1016/S1567-1356(03)00175-2 14654427

[B43] Lachance M. A. Starmer W. T. Rosa C. A. Bowles J. M. Barker J. S. Janzen D. H. (2001). Biogeography of the yeasts of ephemeral flowers and their insects. *FEMS Yeast Res.* 1 1–8. 10.1016/S1567-1356(00)00003-9 12702457

[B44] Lagunes L. Rello J. (2016). Invasive candidiasis: from mycobiome to infection, therapy, and prevention. *Eur. J. Clin. Microbiol. Infect. Dis.* 35 1221–1226. 10.1007/s10096-016-2658-0 27146877

[B45] Lasker B. A. Butler G. Lott T. J. (2006). Molecular genotyping of *Candida parapsilosis* group I clinical isolates by analysis of polymorphic microsatellite markers. *J. Clin. Microbiol.* 44 750–759. 10.1128/JCM.44.3.750 16517850 PMC1393075

[B46] Le S. Q. Gascuel O. (2008). An improved general amino acid replacement matrix. *Mol. Biol. Evol.* 25 1307–1320. 10.1093/molbev/msn067 18367465

[B47] Leipe D. D. Koonin E. V. Aravind L. (2004). STAND, a class of P-Loop NTPases including animal and plant regulators of programmed cell death: multiple, complex domain architectures, unusual phyletic patterns, and evolution by horizontal gene transfer. *J. Mol. Biol.* 343 1–28. 10.1016/j.jmb.2004.08.023 15381417

[B48] Liu X. Q. (2000). Protein-splicing intein: genetic mobility, origin, and evolution. *Annu. Rev. Genet.* 34 61–76. 10.1146/annurev.genet.34.1.61 11092822

[B49] Liu X. Q. Hu Z. (1997). A DnaB intein in *Rhodothermus marinus*: indication of recent intein homing across remotely related organisms. *Proc. Natl. Acad. Sci. U.S.A.* 94 7851–7856. 10.1073/pnas.94.15.7851 9223276 PMC21518

[B50] Liu X. Q. Yang J. (2004). Prp8 intein in fungal pathogens: target for potential antifungal drugs. *FEBS Lett.* 572 46–50. 10.1016/j.febslet.2004.07.016 15304322

[B51] Lo H. J. Köhler J. R. Didomenico B. Loebenberg D. Cacciapuoti A. Fink G. R. (1997). Nonfilamentous *C. albicans* mutants are avirulent. *Cell* 90 939–949. 10.1016/S0092-8674(00)80358-X 9298905

[B52] Lockhart S. R. Iqbal N. Cleveland A. A. Farley M. M. Harrison L. H. Bolden C. B. (2012). Species identification and antifungal susceptibility testing of *Candida* bloodstream isolates from population-based surveillance studies in two U.S. cities from 2008 to 2011. *J. Clin. Microbiol.* 50 3435–3442. 10.1128/JCM.01283-12 22875889 PMC3486211

[B53] Lockhart S. R. Messer S. A. Pfaller M. A. Diekema D. J. (2008). Geographic distribution and antifungal susceptibility of the newly described species *Candida orthopsilosis* and *Candida metapsilosis* in comparison to the closely related species *Candida parapsilosis*. *J. Clin. Microbiol.* 46 2659–2664. 10.1128/JCM.00803-08 18562582 PMC2519489

[B54] Lopez-Martinez R. (2010). Candidosis, a new challenge. *Clin. Dermatol.* 28 178–184. 10.1016/j.clindermatol.2009.12.014 20347660

[B55] Marzluf G. A. (1997). Genetic regulation of nitrogen metabolism in the fungi. *Microbiol. Mol. Biol. Rev.* 61 17–32. 9106362 10.1128/mmbr.61.1.17-32.1997 PMC232598

[B56] Maubon D. Garnaud C. Calandra T. Sanglard D. Cornet M. (2014). Resistance of *Candida* spp. to antifungal drugs in the ICU: where are we now? *Intensive Care Med.* 40 1241–1255. 10.1007/s00134-014-3404-7 25091787

[B57] Merseguel K. Nishikaku A. Rodrigues A. Padovan A. Ferreira R. de Azevedo Melo A. S. (2015). Genetic diversity of medically important and emerging *Candida* species causing invasive infection. *BMC Infect. Dis.* 15:57 10.1186/s12879-015-0793-3 PMC4339437 25887032

[B58] Miletti K. E. Leibowitz M. J. (2000). Pentamidine inhibition of group I intron splicing in *Candida albicans* correlates with growth inhibition. *Antimicrob. Agents Chem.* 44 958–966. 10.1128/AAC.44.4.958-966.2000 PMC89798 10722497

[B59] Miraula M. Enculescu C. Schenk G. Mitić N. (2015). Applications of splicing-promoting proteins. *Am. J. Mol. Biol.* 5 42–56. 10.4236/ajmb.2015.52005

[B60] Mita P. Boeke J. D. (2016). How retrotransposons shape genome regulation. *Curr. Opin. Genet. Dev.* 37 90–100. 10.1016/j.gde.2016.01.001 26855260 PMC4914423

[B61] Nagasaki K. Shirai Y. Tomaru Y. Nishida K. Pietrokovski S. (2005). Algal viruses with distinct intraspecies host specificities include identical intein elements. *Appl. Environ. Microbiol.* 71 3599–3607. 10.1128/AEM.71.7.3599-3607.2005 16000767 PMC1169056

[B62] Nakase T. (1971). New species resembling of yeasts *Candida krusei* (cast.) Berkhout. *J. Gen. Appl. Microbiol.* 398 383–398. 10.2323/jgam.17.383

[B63] Nakase T. Suzuki M. Takashima M. Miyakawa Y. Kagaya K. Fukazawa Y. (1994). *Candida sojae*, a new species of yeast isolated from an extraction process of water soluble substances of defatted soubean flakes. *J. Gen. Appl. Microbiol.* 40 161–169. 10.2323/jgam.40.161

[B64] Nelson N. Perzov N. Cohen A. Hagai K. Padler V. Nelson H. (2000). The cellular biology of proton-motive force generation by V-ATPases. *J. Exp. Biol.* 203 89–95. 10600677 10.1242/jeb.203.1.89

[B65] Novikova O. Jayachandran P. Kelley D. S. Morton Z. Merwin S. Topilina N. I. (2015). Intein clustering suggests functional importance in different domains of life. *Mol. Biol. Evol.* 33 783–799. 10.1093/molbev/msv271 26609079 PMC4760082

[B66] Novikova O. Topilina N. Belfort M. (2014). Enigmatic distribution, evolution, and function of Inteins. *J. Biol. Chem.* 289 14490–14497. 10.1074/jbc.R114.548255 24695741 PMC4031506

[B67] Nucci M. Queiroz-Telles F. Tobón A. M. Restrepo A. Colombo A. L. (2010). Epidemiology of opportunistic fungal infections in Latin America. *Clin. Infect. Dis.* 51 561–570. 10.1086/655683 20658942

[B68] Ochsner U. A. Sun X. Jarvis T. Critchley I. Janjic N. (2007). Aminoacyl-tRNA synthetases: essential and still promising targets for new anti-infective agents. *Expert. Opin. Investig. Drug* 16 573–593. 10.1517/13543784.16.5.573 17461733

[B69] O’Donoghue P. Luthey-Schulten Z. (2003). On the evolution of structure in aminoacyl-tRNA synthetases. *Microbiol. Mol. Biol. Rev.* 67 550–573. 10.1128/MMBR.67.4.550 14665676 PMC309052

[B70] Okuda Y. Sasaki D. Nogami S. Kaneko Y. Ohya Y. Anraku Y. (2003). Occurrence, horizontal transfer and degeneration of VDE intein family in Saccharomycete yeasts. *Yeast* 20 563–573. 10.1002/yea.984 12734795

[B71] Paramythiotou E. Frantzeskaki F. Flevari A. Armaganidis A. Dimopoulos G. (2014). Invasive fungal infections in the ICU: how to approach, how to treat. *Molecules* 19 1085–1119. 10.3390/molecules19011085 24445340 PMC6271196

[B72] Paulus H. (2003). Inteins as targets for potential antimycobacterial drugs. *Front. Biosci.* 1:1157–1165. 10.2741/1195 12957838

[B73] Paulus H. (2007). Protein splicing inhibitors as a new class of antimycobacterial agents. *Drugs Fut.* 32 973–984. 10.1358/dof.2007.032.11.1140690

[B74] Perler F. B. (2002). InBase: the intein database. *Nucleic Acids Res.* 30 383–384. 10.1093/nar/30.1.383 11752343 PMC99080

[B75] Perler F. B. (2005). Protein splicing mechanisms and applications. *IUBMB Life* 57 469–476. 10.1080/15216540500163343 16081367

[B76] Pfaller M. A. Andes D. R. Diekema D. J. Horn D. L. Reboli A. C. Rotstein C. (2014). Epidemiology and outcomes of invasive candidiasis due to non-albicans species of *Candida* in 2,496 patients: data from the Prospective Antifungal Therapy (PATH) registry 2004–2008. *PLoS ONE* 9:e101510 10.1371/journal.pone.0101510 PMC4081561 24991967

[B77] Pfaller M. A. Chaturvedi V. Diekema D. J. Ghannoum M. A. Holliday N. M. Killian S. B. (2012). Comparison of the sensititre YeastOne colorimetric antifungal panel with CLSI microdilution for antifungal susceptibility testing of the echinocandins against *Candida* spp., using new clinical breakpoints and epidemiological cutoff values. *Diagn. Microbiol. Infect. Dis.* 73 365–368. 10.1016/j.diagmicrobio.2012.05.008 22726528

[B78] Pfaller M. A. Diekema D. J. (2007). Epidemiology of invasive candidiasis: a persistent public health problem. *Clin. Microbiol. Rev.* 20 133–163. 10.1128/CMR.00029-06 17223626 PMC1797637

[B79] Posey K. L. Koufopanou V. Burt A. Gimble F. S. (2004). Evolution of divergent DNA recognition specificities in VDE homing endonucleases from two yeast species. *Nucleic Acids Res.* 32 3947–3956. 10.1093/nar/gkh734 15280510 PMC506816

[B80] Poulter R. T. M. Goodwin T. J. D. Butler M. I. (2007). The nuclear-encoded inteins of fungi. *Fungal Genet. Biol.* 44 153–179. 10.1016/j.fgb.2006.07.012 17046294

[B81] Prandini T. H. R. Theodoro R. C. Bruder-Nascimento A. C. M. O. Scheel C. M. Bagagli E. (2013). Analysis of inteins in the *Candida parapsilosis* complex for simple and accurate species identification. *J. Clin. Microbiol.* 51 2830–2836. 10.1128/JCM.00981-13 23784117 PMC3754621

[B82] Pryszcz L. P. Németh T. Gácser A. Gabaldón T. (2014). Genome comparison of *Candida orthopsilosis* clinical strains reveals the existence of hybrids between two distinct subspecies. *Genome Biol. Evol.* 6 1069–1078. 10.1093/gbe/evu082 24747362 PMC4040990

[B83] Pryszcz L. P. Németh T. Saus E. Ksiezopolska E. Hegedűsová E. Nosek J. (2015). The genomic aftermath of hybridization in the opportunistic pathogen *Candida metapsilosis*. *PLoS Genet.* 11:e1005626 10.1371/journal.pgen.1005626 PMC4627764 26517373

[B84] Quiles-Melero I. García-Rodríguez J. Gómez-López A. Mingorance J. (2012). Evaluation of matrix-assisted laser desorption/ionisation time-of-flight (MALDI-TOF) mass spectrometry for identification of *Candida parapsilosis, C. orthopsilosis* and *C. metapsilosis*. *Eur. J. Clin. Microbiol. Infect. Dis.* 31 67–71. 10.1007/s10096-011-1277-z 21547602

[B85] Ramachandra S. Linde J. Brock M. Guthke R. Hube B. Brunke S. (2014). Regulatory networks controlling nitrogen sensing and uptake in *Candida albicans*. *PLoS ONE* 9:e92734 10.1371/journal.pone.0092734 PMC3961412 24651113

[B86] Richardson M. Lass-Flörl C. (2008). Changing epidemiology of systemic fungal infections. *Clin. Microbiol. Infect.* 14 5–24. 10.1111/j.1469-0691.2008.01978.x 18430126

[B87] Ruan S.-Y. Chien J.-Y. Hou Y.-C. Hsueh P.-R. (2010). Catheter-related fungemia caused by *Candida* intermedia. *Int. J. Infect. Dis.* 14 147–149. 10.1016/j.ijid.2009.03.015 19497773

[B88] Sai S. Holland L. M. McGee C. F. Lynch D. B. Butler G. (2011). Evolution of mating within the *Candida parapsilosis* species group. *Eukaryot. Cell* 10 578–587. 10.1128/EC.00276-10 21335529 PMC3127640

[B89] Sardi J. C. O. Scorzoni L. Bernardi T. Fusco-Almeida A. M. Mendes Giannini M. J. S. (2013). *Candida* species: current epidemiology, pathogenicity, biofilm formation, natural antifungal products and new therapeutic options. *J. Med. Microbiol.* 62 10–24. 10.1099/jmm.0.045054-0 23180477

[B90] Satish Kumar R. Ramesh S. (2014). Novel intein-containing DNA specific primers for rapid identification of *Candida glabrata* using Real-Time PCR assays. *J. Med. Mycol.* 24 337–340. 10.1016/j.mycmed.2014.08.002 25282343

[B91] Saville S. P. Lazzell A. L. Bryant A. P. Fretzen A. Monreal A. Solberg E. O. (2006). Inhibition of filamentation can be used to treat disseminated candidiasis. *Antimicrob. Agents Chemother.* 50 3312–3316. 10.1128/AAC.00628-06 17005810 PMC1610055

[B92] Sharp P. M. Tuohy T. M. F. Mosurski K. R. (1986). Codon usage in yeast: cluster analysis clearly differentiates highly and lowly expressed genes. *Nucleic Acids Res.* 14 5125–5143. 10.1093/nar/14.13.5125 3526280 PMC311530

[B93] Souza A. C. R. Ferreira R. C. Gonçalves S. S. Quindós G. Eraso E. Bizerra F. C. (2012). Accurate identification of *Candida parapsilosis* (sensu lato) by use of mitochondrial DNA and real-time PCR. *J. Clin. Microbiol.* 50 2310–2314. 10.1128/JCM.00303-12 22535986 PMC3405582

[B94] Spampinato C. Leonardi D. (2013). *Candida* infections, causes, targets, and resistance mechanisms: traditional and alternative antifungal agents. *Biomed Res. Int.* 2013:204237 10.1155/2013/204237 PMC3708393 23878798

[B95] Steuer S. Pingoud V. Pingoud A. Wende W. (2004). Chimeras of the homing endonuclease PI-Scel and the homologous *Candida tropicalis* intein: a study to explore the possibility of exchanging DNA-binding modules to obtain highly specific endonucleases with altered specificity. *Chembiochem* 5 206–213. 10.1002/cbic.200300718 14760742

[B96] Sudbery P. E. (2011). Growth of *Candida albicans* hyphae. *Nat. Rev. Microbiol.* 9 737–748. 10.1038/nrmicro2636 21844880

[B97] Swithers K. Senejani A. Fournier G. Gogarten J. P. (2009). Conservation of intron and intein insertion sites: implications for life histories of parasitic genetic elements. *BMC Evol. Biol.* 9:303 10.1186/1471-2148-9-303 PMC2814812 20043855

[B98] Tamura K. Stecher G. Peterson D. Filipski A. Kumar S. (2013). MEGA6: molecular evolutionary genetics analysis version 6.0. *Mol. Biol. Evol.* 30 2725–2729. 10.1093/molbev/mst197 24132122 PMC3840312

[B99] Tavanti A. Davidson A. D. Gow N. A. R. Maiden M. C. J. Odds F. C. (2005). *Candida parapsilosis* groups II and III. *J. Clin. Microbiol.* 43 284–292. 10.1128/JCM.43.1.284 15634984 PMC540126

[B100] Theodoro R. C. Bagagli E. (2009). Inteins in pathogenic fungi: a phylogenetic tool and perspectives for therapeutic applications. *Mem. Inst. Oswaldo Cruz* 104 497–504. 10.1590/S0074-02762009000300017 19547879

[B101] Theodoro R. C. Bagagli E. Oliveira C. (2008). Phylogenetic analysis of PRP8 intein in *Paracoccidioides brasiliensis* species complex. *Fungal Genet. Biol.* 45 1284–1291. 10.1016/j.fgb.2008.07.003 18672080

[B102] Theodoro R. C. Scheel C. M. Brandt M. E. Kasuga T. Bagagli E. (2013). PRP8 intein in cryptic species of *Histoplasma capsulatum*: evolution and phylogeny. *Infect. Genet. Evol.* 18 174–182. 10.1016/j.meegid.2013.05.001 23665464

[B103] Theodoro R. C. Volkmann G. Liu X. Q. Bagagli E. (2010). PRP8 intein in Ajellomycetaceae family pathogens: sequence analysis, splicing evaluation and homing endonuclease activity. *Fungal Genet. Biol.* 48 80–91. 10.1016/j.fgb.2010.07.010 20682355

[B104] Topilina N. I. Green C. M. Jayachandran P. Kelley D. S. Stanger M. J. Lyn C. (2015a). SufB intein of *Mycobacterium tuberculosis* as a sensor for oxidative and nitrosative stresses. *Proc. Natl. Acad. Sci. U.S.A.* 112 10348–10353. 10.1073/pnas.1512777112 26240361 PMC4547236

[B105] Topilina N. I. Novikova O. Stanger M. Banavali N. K. Belfort M. (2015b). Post-translational environmental switch of RadA activity by extein – intein interactions in protein splicing. *Nucleic Acids Res.* 43 6631–6648. 10.1093/nar/gkv612 26101259 PMC4513877

[B106] Tran A. Alby K. Kerr A. Jones M. Gilligan P. H. (2015). Cost savings realized by implementation of routine microbiological Identification by matrix-assisted laser desorption ionization–time of flight mass spectrometry. *J. Clin. Microbiol.* 53 2473–2479. 10.1128/JCM.00833-15 25994167 PMC4508454

[B107] Valenzuela L. Ballario P. Aranda C. Filetici P. González A. (1998). Regulation of expression of GLT1, the gene encoding glutamate synthase in *Saccharomyces cerevisiae*. *J. Bacteriol.* 180 3533–3540. 9657994 10.1128/jb.180.14.3533-3540.1998 PMC107319

[B108] Vaughan-Martini A. Kurtzman C. P. Meyer S. A. O’Neill E. B. (2005). Two new species in the *Pichia guilliermondii* clade: *Pichia caribbica* sp. nov., the ascosporic state of *Candida fermentati*, and *Candida carpophila* comb. nov. *FEMS Yeast Res.* 5 463–469. 10.1016/j.femsyr.2004.10.008 15691751

[B109] Vega-Alvarado L. Gómez-Angulo J. Escalante-García Z. Grande R. Gschaedler-Mathis A. Amaya-Delgado L. (2015). High-quality draft genome sequence of *Candida apicola* NRRL Y-50540. *Genome Announc.* 3 e00437-15 10.1128/genomeA.00437-15 PMC4463513 26067948

[B110] Volkmann G. Iwaı H. (2010). Protein trans -splicing and its use in structural biology?: opportunities and limitations. *Mol. Biosyst.* 6 2110–2121. 10.1039/c0mb00034e 20820635

[B111] Vondenhoff G. H. M. Van Aerschot A. (2011). Aminoacyl-tRNA synthetase inhibitors as potential antibiotics. *Eur. J. Med. Chem.* 46 5227–5236. 10.1016/j.ejmech.2011.08.049 21968372

[B112] Wang H. Xiao M. Chen S. C. A. Kong F. Sun Z. Y. Liao K. (2012). In Vitro susceptibilities of yeast species to fluconazole and voriconazole as determined by the 2010 National China Hospital invasive fungal surveillance net (CHIF-NET) study. *J. Clin. Microbiol.* 50 3952–3959. 10.1128/JCM.01130-12 23035204 PMC3502960

[B113] Whelan S. Goldman N. (2001). A general empirical model of protein evolution derived from multiple protein families using a maximum-likelihood approach. *Mol. Biol. Evol.* 18 691–699. 10.1093/oxfordjournals.molbev.a003851 11319253

[B114] Wisplinghoff H. Bischoff T. Tallent S. M. Seifert H. Wenzel R. P. Edmond M. B. (2004). Nosocomial bloodstream infections in US hospitals: analysis of 24,179 cases from a prospective nationwide surveillance study. *Clin. Infect. Dis.* 39 309–317. 10.1086/421946 15306996

[B115] Wolfe K. H. Riccombeni A. Vidanes G. Proux-we E. Butler G. (2012). Sequence and analysis of the genome of the pathogenic yeast *Candida orthopsilosis*. *PLoS ONE* 7:e35750 10.1371/journal.pone.0035750 PMC3338533 22563396

[B116] Wood D. W. Wu W. Belfort G. Derbyshire V. Belfort M. (1999). A genetic system yields self-cleaving inteins for bioseparations. *Nat. Biotechnol.* 17 889–892. 10.1038/12879 10471931

[B117] Yapar N. (2014). Epidemiology and risk factors for invasive candidiasis. *Ther. Clin. Risk Manag.* 10 95–105. 10.2147/TCRM.S40160 24611015 PMC3928396

[B118] Yun Y. H. Suh D. Y. Yoo H. D. Oh M. H. Kim S. H. (2015). Yeast associated with the ambrosia beetle, *Platypus* koryoensis, the pest of oak trees in Korea. *Mycobiology* 43 458–466. 10.5941/MYCO.2015.43.4.458 26839506 PMC4731651

[B119] Zhang L. Zheng Y. Callahan B. Belfort M. Liu Y. (2010). Cisplatin inhibits protein splicing, suggesting inteins as therapeutic targets in mycobacteria. *J. Biol. Chem.* 286 1277–1282. 10.1074/jbc.M110.171124 21059649 PMC3020735

[B120] Zhang Y.-Q. Rao R. (2010). Beyond ergosterol: linking pH to antifungal mechanisms. *Virulence* 1 551–554. 10.4161/viru.1.6.13802 21178501

