# Supplementary material for: Evolution and Application of Inteins in Candida species: A Review
Source: Front Microbiol. 2016 Oct 10;7:1585. doi: 10.3389/fmicb.2016.01585 (PMC5056185; doi:10.3389/fmicb.2016.01585)
Supplement: Supplementary file 1 [file Table_1.DOC]

**Supplementary Table 1:** Analyzed genomes from NCBI WGS and MycoCosm databases.

| **NCBI WGS** | | | |
| --- | --- | --- | --- |
| **Organism** | **Strain** | **BioProject** | **BioSample** |
| *C. albicans* | 12C | PRJNA75209 | SAMN00767974 |
|  | 19F | PRJNA75221 | SAMN01048008 |
|  | 3153A | PRJNA165021 | SAMN00974104 |
|  | A123 | PRJNA165033 | SAMN00974110 |
|  | A155 | PRJNA165035 | SAMN00974111 |
|  | A20 | PRJNA165025 | SAMN00974106 |
|  | A203 | PRJNA165039 | SAMN00974113 |
|  | A48 | PRJNA165027 | SAMN00974107 |
|  | A67 | PRJNA165029 | SAMN00974108 |
|  | A84 | PRJNA165037 | SAMN00974112 |
|  | A92 | PRJNA165031 | SAMN00974109 |
|  | CA529L | PRJNA200311 | SAMN02058435 |
|  | CA6 | PRJNA120431 | SAMN03164130 |
|  | CHN1 | PRJNA165023 | SAMN00974105 |
|  | GC75 | PRJNA75223 | SAMN00767984 |
|  | L26 | PRJNA75211 | SAMN01048004 |
|  | P34048 | PRJNA75229 | SAMN01048010 |
|  | P37005 | PRJNA75217 | SAMN01048006 |
|  | P37037 | PRJNA75231 | SAMN01048011 |
|  | P37039 | PRJNA75233 | SAMN00767975 |
|  | P57055 | PRJNA75239 | SAMN01048013 |
|  | P57072 | PRJNA75227 | SAMN00767978 |
|  | P60002 | PRJNA75219 | SAMN01048007 |
|  | P75010 | PRJNA75235 | SAMN00769059 |
|  | P75016 | PRJNA75237 | SAMN01048012 |
|  | P75063 | PRJNA75241 | SAMN01048014 |
|  | P76055 | PRJNA75243 | SAMN01048016 |
|  | P76067 | PRJNA75245 | SAMN01048017 |
|  | P78042 | PRJNA75247 | SAMN01048015 |
|  | P78048 | PRJNA75225 | SAMN01048009 |
|  | P87 | PRJNA75215 | SAMN00767982 |
|  | P94015 | PRJNA75213 | SAMN01048005 |
|  | SC5314 | PRJNA10701 | SAMN02953594 |
|  | SC5314 | PRJNA120009 | SAMN01041717 |
|  | SC5314 | PRJNA191536 | SAMN01041717 |
|  | SC5314 | PRJNA14005 |  |
|  | WO-1 | PRJNA16373 | SAMN02953609 |
| *C. apicola* | NRRL Y-50540 | PRJNA278776 | SAMN03431278 |
| *C. auris* | VPCI 479/P/13 | PRJEB9463 | SAMEA3428689 |
|  | 6684 | PRJNA267757 | SAMN03200169 |
| *C. boidinii* | JCM 9604 | PRJDB3623 | SAMD00028343 |
|  | GF002 | PRJNA299882 | SAMN04216991 |
| *C. bracarensis* | CBS 10154 | PRJEB145 | SAMEA3146302 |
| *C. carpophila* | JCM 9396 | PRJDB3618 | SAMD00028338 |
| *C. castellii* | CBS 4332 | PRJEB147 | SAMEA3146276 |
| *C. dubliniensis* | UOB319 | PRJNA221141 | SAMN02593544 |
|  | UOB320 | PRJNA221141 | SAMN02593545 |
| *C. glabrata* | 1A | PRJNA297263 | SAMN04121073 |
|  | 2B | PRJNA297263 | SAMN04121076 |
|  | 2A | PRJNA297263 | SAMN04121075 |
|  | 1B | PRJNA297263 | SAMN04121074 |
|  | 3B | PRJNA297263 | SAMN04121078 |
|  | 3A | PRJNA297263 | SAMN04121077 |
|  | CCTCC M202019 | PRJNA222546 | SAMN02981566 |
| *C. homilentoma* | JCM 1507 | PRJDB3610 | SAMD00028329 |
| *C. infanticola* | DS02 | PRJNA318722 | SAMN04866663 |
| *C. intermedia* | JCM 1607 | PRJDB3612 | SAMD00028331 |
| *C. maltosa* | XU316 | PRJNA184737 | SAMN02981501 |
| *C. metapsilosis* | CANME | PRJEB1698 | SAMEA3158484 |
| *C. nivariensis* | CBS 9983 | PRJEB148 | SAMEA3146320 |
| *C. orthopsilosis* | MCO456 | PRJEB4430 | SAMEA3146311 |
|  | AY2 | PRJNA171019 | SAMN02981454 |
| *C. parapsilosis* | CBS1954 | PRJEB5470 | SAMEA3146297 |
|  | CBS6318 | PRJEB5471 | SAMEA3146268 |
|  | GA1 | PRJEB1685 | SAMEA3146298 |
|  | CDC317 | PRJEA32889 | SAMEA2272681 |
| *C. sojae* | GF41 | PRJNA299010 | SAMN04192112 |
| *C. sorboxylosa* | JCM 1536 | PRJDB3611 | SAMD00028330 |
| *Candida sp.* | LDI48194 | PRJNA282657 | SAMN03573713 |
| *C. succiphila* | JCM 9445 | PRJDB3619 | SAMD00028339 |
| *C. tenuis* | ATCC 10573 | PRJNA33673 | SAMN00715317 |
| *C. tropicalis* | 121 | PRJNA238598 | SAMN02650963 |
|  | MYA-3404 | PRJNA13675 | SAMN02953608 |
| *C. versatilis* | JCM 5958 | PRJDB3712 | SAMD00028433 |
| *Lodderomyces elongisporus* | NRRL YB-4239 | PRJNA19611 | SAMN02953647 |
| *C. colliculosa* | CBS 1146 | PRJEA70971 | SAMEA2272433 |
| *Pichia kudriavzevii*  (*C. krusei*) | M12 | PRJNA171021 | SAMN02981439 |
| **MycoCosm** | | | |
| **Organism** | **Strain** | **JGI Project Id** | **NCBI Project Id** |
| *C. arabinofermentans* | NRRL YB-2248 | 403652 | 207879 |
| *C. tanzawaensis* | NRRL Y-17324 | 403655 | 207878 |
| *C. tenuis* | NRRL Y-1498 | 400298 | 33673 |
| *C. caseinolytica* | Y-17796 | 402672 | 76687 |
