# Supplementary material for: Evolution and Application of Inteins in Candida species: A Review
Source: Front Microbiol. 2016 Oct 10;7:1585. doi: 10.3389/fmicb.2016.01585 (PMC5056185; doi:10.3389/fmicb.2016.01585)
Supplement: Supplementary file 2 [file Image_1.PDF]

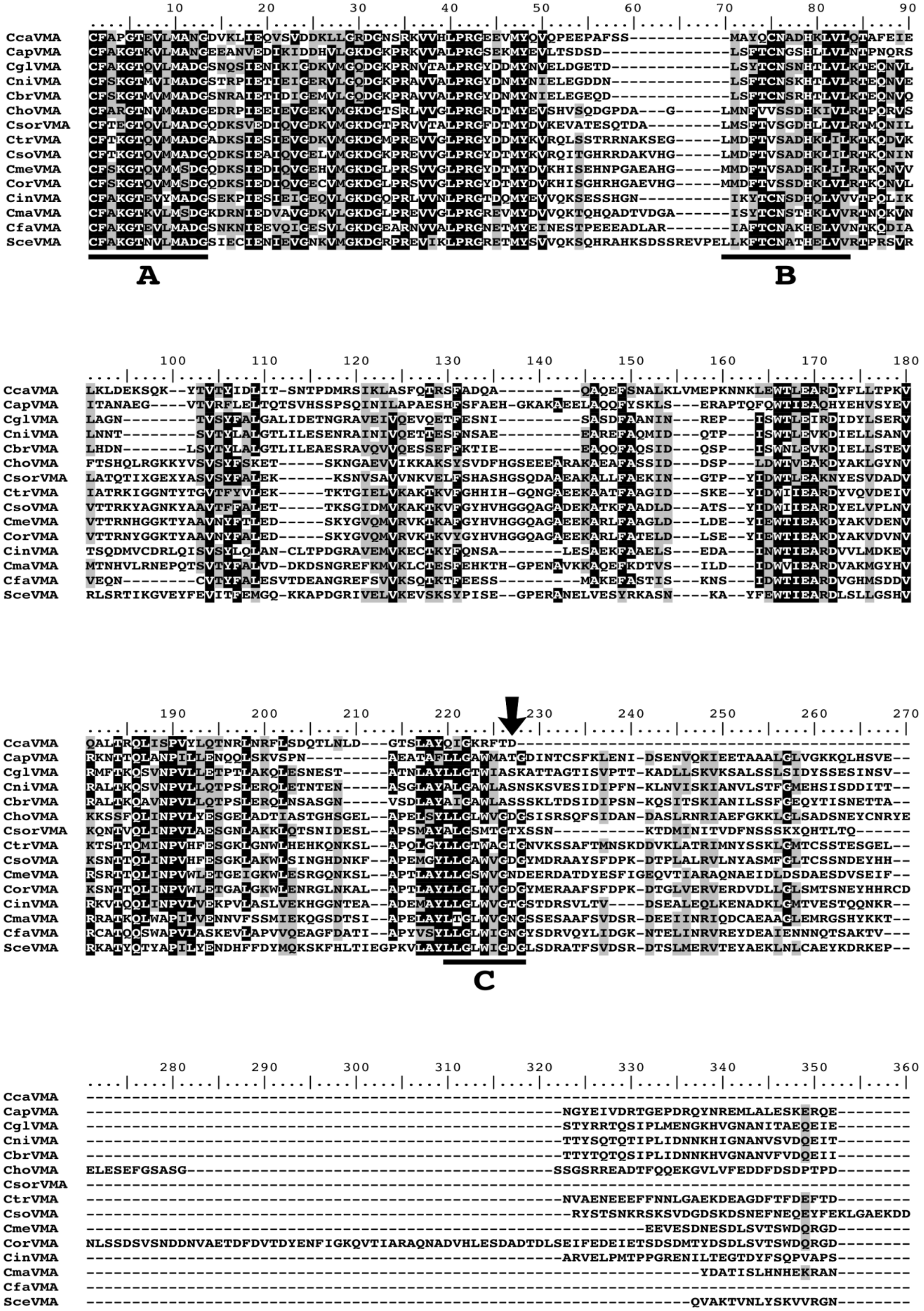
CcaVMA
CapVMA
CglVMA
CniVMA
CbrVMA
ChoVMA
CsorVMA
CtrVMA
CsoVMA
CmeVMA
CorVMA
CinVMA
CmaVMA
CfaVMA
SceVMA
A
B
C

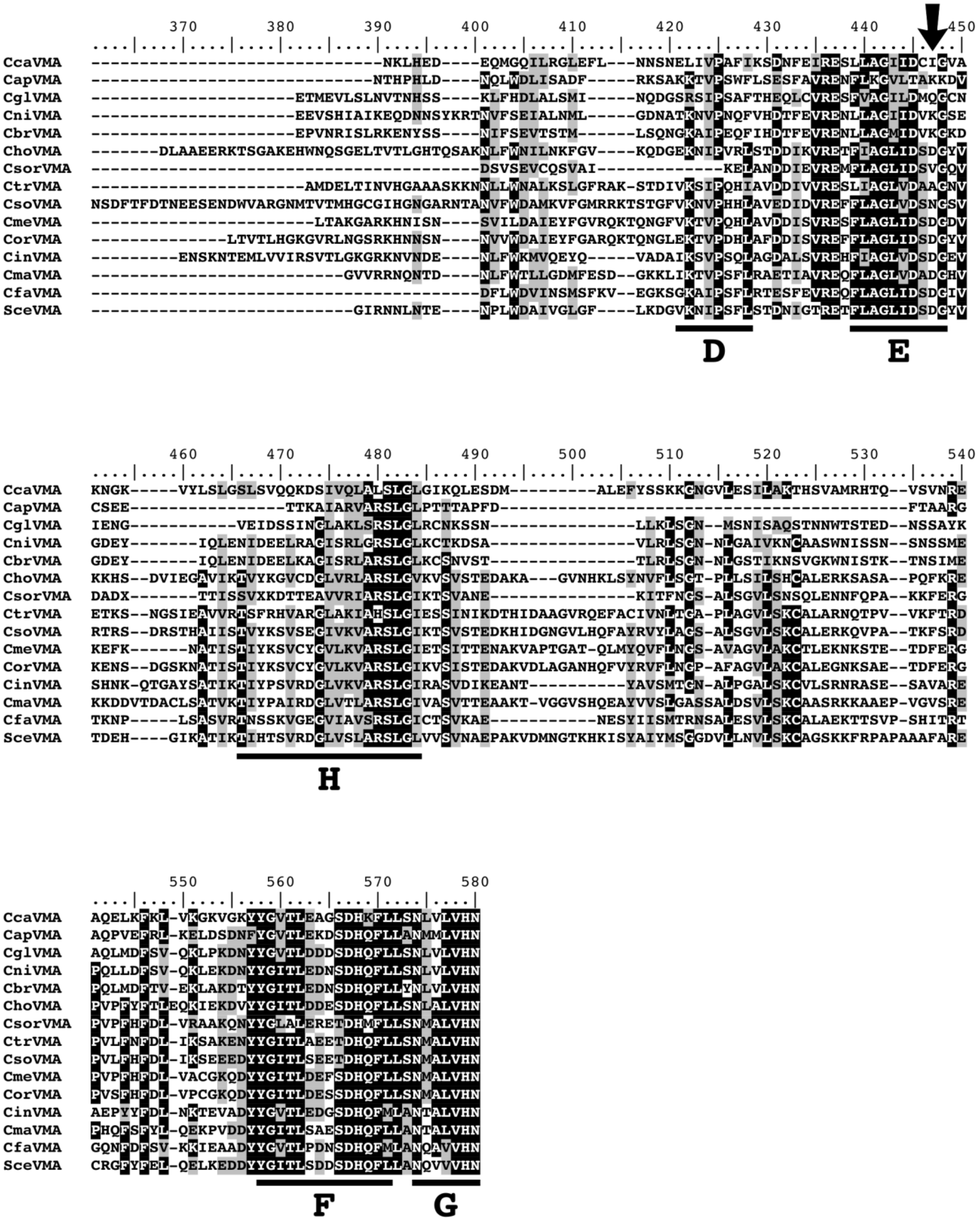

Supplementary Figure 1: amino acids sequence alignment, by Muscle, of VMA intein from different *Candida* species. The location of conserved motifs from splicing (A, B, F and G) and HE (C, D, E and H) domains are indicated. The arrows indicate the position of the two essential aspartic acids according to SceVMA intein.
