# Supplementary figures and images for: Evolution and Application of Inteins in Candida species: A Review
Source: Front Microbiol. 2016 Oct 10;7:1585. doi: 10.3389/fmicb.2016.01585 (PMC5056185; doi:10.3389/fmicb.2016.01585)

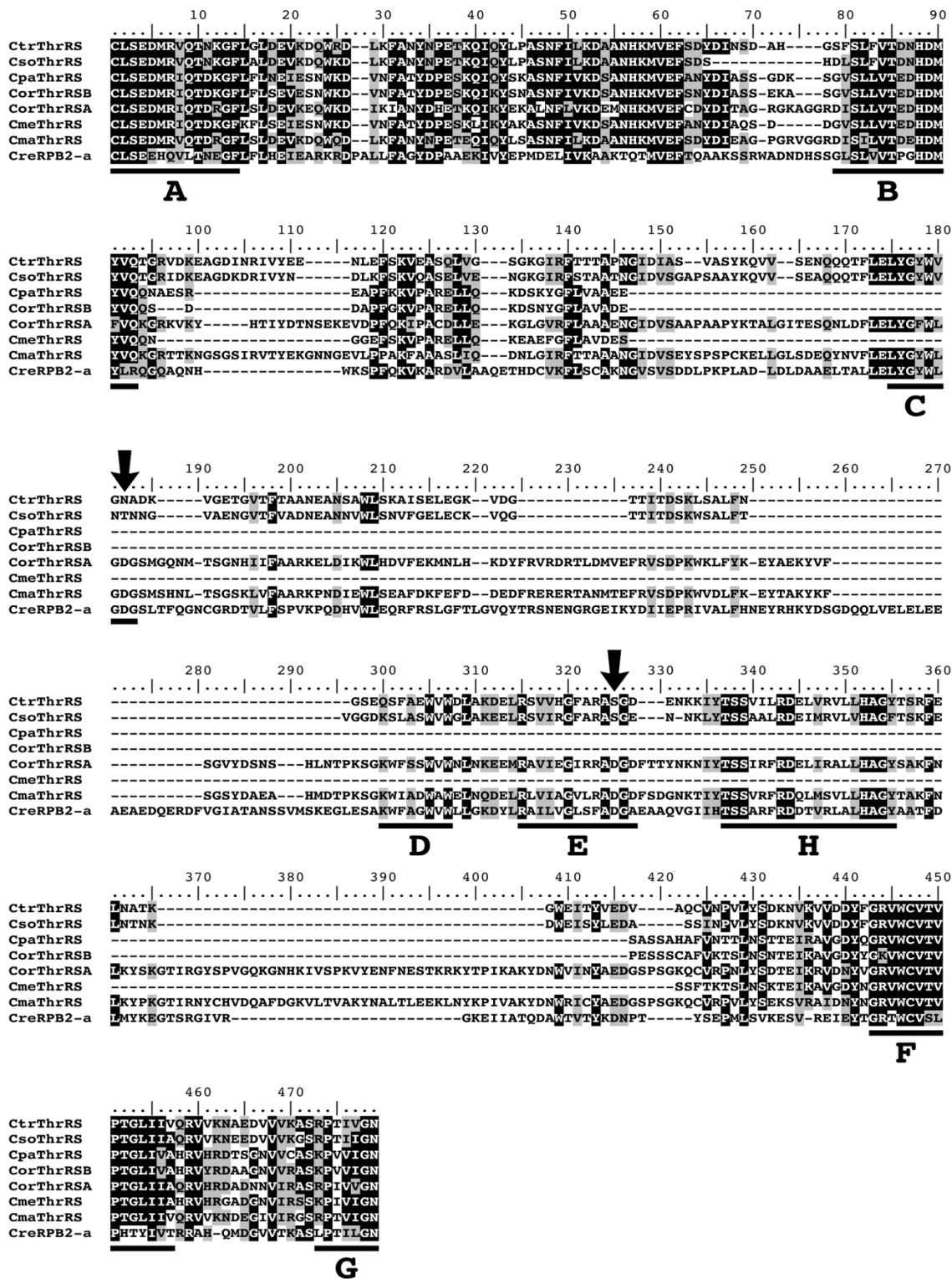

Supplement: Supplementary file 3 [file Image_2.PDF]
