# Supplementary material for: Evolution and Application of Inteins in Candida species: A Review
Source: Front Microbiol. 2016 Oct 10;7:1585. doi: 10.3389/fmicb.2016.01585 (PMC5056185; doi:10.3389/fmicb.2016.01585)
Supplement: Supplementary file 5 [file Image_4.PDF]

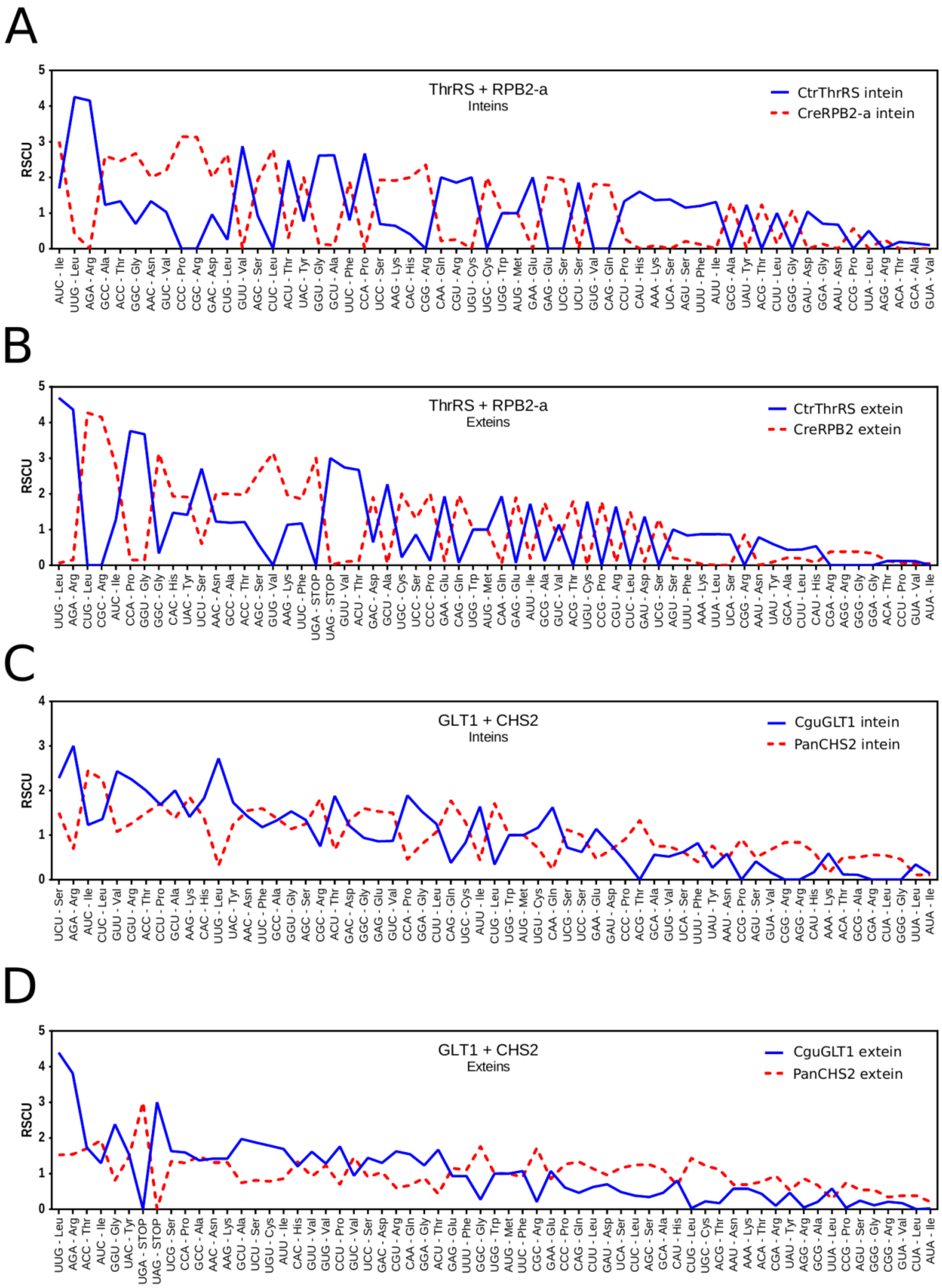

Supplementary Figure 4: Graphic view of RSCU values for codon usage in ThrRS, GLT1, RBP2 and CHS2 inteins and their respective exteins. Comparison between: **(A)** RSCU values for CtrThrRS and CreRPB2-a inteins; **(B)** RSCU values for *THRRS* and *RPB2* genes of *C. tropicalis* and *C. reinhardtii*, respectively without intein; **(C)** RSCU values for CguGLT1 and PanCHS2 inteins **(D)** RSCU values for *GLT1* and *CHS2* genes of *C. tropicalis* and *P. anserina*, respectively, without intein.
